# Identification of a nuclear transcriptional module associated with ERα and E2F/FOXM1 signaling across PAM50 breast cancer subtypes

**DOI:** 10.3389/fonc.2026.1874614

**Published:** 2026-08-05

**Authors:** Ana Jazmín Dozal-Luna, Sandra K. Santuario-Facio, Servando Cardona-Huerta, Gabriela Sofía Gómez-Macías, Claudia Rangel-Escareno, Saúl Lira-Albarrán, Liliana Gómez-Flores-Ramos, Rocío Ortiz-López

**Affiliations:** 1 Escuela de Medicina y Ciencias de la Salud, Tecnológico de Monterrey, Monterrey, Mexico; 2 Laboratorio Clínico, Hospital Zambrano Hellion, TecSalud, Tecnológico de Monterrey, San Pedro Garza García, Mexico; 3 Centro de Cáncer de Mama, Hospital Zambrano Hellion, TecSalud, Tecnológico de Monterrey, San Pedro Garza García, Mexico; 4 Departamento de Patología, Hospital Zambrano Hellion, TecSalud, Tecnológico de Monterrey, San Pedro Garza García, Mexico; 5 Departamento de Patología, Facultad de Medicina, Universidad Autónoma de Nuevo León, Monterrey, Mexico; 6 Genómica Computacional y Biología Integrativa, Instituto Nacional de Medicina Genómica, Ciudad de México, Mexico; 7 Escuela de Ingeniería y Ciencias, Tecnológico de Monterrey, Monterrey, Mexico; 8 Microlab Genomics, Laboratorio Microlab, Tegucigalpa, Honduras; 9 Centro de Investigación en Salud Poblacional, Instituto Nacional de Salud Pública, Secretaría de Ciencia, Humanidades, Tecnología e Innovación (SECIHTI), Cuernavaca, Mexico; 10 Proyecto oriGen, Tecnológico de Monterrey, Monterrey, Mexico

**Keywords:** biomarkers, breast cancer, estrogen receptor, molecular classification, proliferation, signaling pathway

## Abstract

**Background:**

Breast cancer is the most prevalent and lethal cancer among women worldwide. Although classification based on ERα, PR, and HER2 status guides treatment decisions, misclassification remains a challenge due to the complexity of ERα-associated transcriptional network. Identifying biomarkers that better reflect ERα activity may improve tumor stratification and therapeutic decision-making. We hypothesized that an ERα-related transcriptional framework could improve molecular characterization of breast cancer subtypes.

**Methods:**

Clinicopathological features were evaluated in relation to PAM50 subtypes. Gene expression profiles from fresh breast tumor biopsies (n=65) were analyzed and integrated with ERα-related genes identified through literature review and pathway databases (KEGG, WikiPathways, Reactome, and IPA). Differentially expressed genes among subtypes were used to construct a nuclear module for functional enrichment and protein interaction analyses. Selected genes were evaluated by RT-qPCR in breast cancer cell lines. The reproducibility of the module expression was assessed in the independent public cohort GSE18229. Correlation analyses were performed to evaluate associations between module genes and *MKI67*/Ki-67 expression.

**Results:**

Menopausal status showed a significant association with PAM50 subtypes. Among 647 ERα-related genes, a 34-gene nuclear module was identified as a highly interconnected network. Genes enriched in LumB, HER2-enriched, and basal and downregulated in LumA and normal-like subtypes were mainly associated with proliferation and cell cycle regulation (*CCNE1, E2F2, FOXM1, PLK1*, and *SKP2*). The ERα–E2F/FOXM1 axis emerged as a potential regulator of the transcriptional module. RT-qPCR supported the expression of selected genes *E2F2, PAK2*, and *PARP1*. The module showed reproducible expression patterns in an independent cohort. Correlation analysis revealed proliferation and chromatin-related genes positively and hormone-related genes negatively associated with *MKI67*/Ki-67, identifying the most significant associations in the study.

**Conclusion:**

This 34-gene module represents an interconnected transcriptional program enriched in E2F/FOXM1 signaling and ERα-associated processes. The integrated analysis of genes involved in hormone signaling, proliferation, genome regulation, and cancer-related processes may reveal subtype-specific expression patterns reflecting ERα activity. We propose the 34-gene panel as a candidate transcriptional framework that may provide insights into BC subtype-discrimination. Further validation in larger cohorts and additional experimental validation are required to determine its potential utility as a biomarker signature for breast cancer stratification.

## 1 Introduction

Breast cancer (BC) remains the most frequently diagnosed malignancy among women worldwide (1). Tumor classification is central to guiding treatment decisions and is commonly based on either gene expression profiling (e.g., PAM50) or immunohistochemical (IHC) assessment of three key markers: estrogen receptor alpha (ERα), progesterone receptor (PR), and human epidermal growth factor receptor 2 (HER2). These approaches define major BC subtypes, including luminal A (LumA), luminal B (LumB), HER2-enriched, and triple-negative (TN) tumors, each associated with distinct biological and clinical characteristics (2).

Despite its widespread use, this classification framework relies on a limited number of markers and may not fully capture the underlying transcriptional complexity of breast tumors. Among these markers, ERα (encoded by *ESR1*) plays a central role, being expressed in approximately 70% of BC cases and serving as a primary determinant of therapeutic strategies (3). ERα signaling involves a complex network of transcription factors and downstream targets regulating key oncogenic processes, including proliferation, differentiation, and apoptosis (4). However, the full extent of ERα–mediated transcriptional regulation remains incompletely characterized, limiting our ability to accurately interpret its functional activity across tumor subtypes.

This limitation has important clinical implications. Accurate tumor classification directly influences treatment selection and patient outcomes (5). For example, although luminal A and luminal B tumors are both ER-positive, only a subset of luminal B patients derive benefit from adjuvant chemotherapy (6). Similarly, misidentification of luminal tumors as TN tumors (due to low-ERα protein expression) and vice versa is another current challenge to overcome (7). Misclassification may therefore lead to suboptimal treatment decisions, such as withholding endocrine therapy or administering unnecessary chemotherapy. Notably, tumor misclassification has been reported in approximately 10–30% of BC cases (8, 9), highlighting the need for more precise and biologically informed classification strategies.

In this study, we sought to characterize transcriptional patterns that more accurately reflect ERα activity across BC subtypes. Through an integrative analysis approach, we identified a 34-gene nuclear module associated with ERα signaling and subtype-specific biological processes, in which the E2F and FOXM1 transcriptional network emerged as relevant components. Given their established roles in cell cycle regulation and proliferation in BC (10–12), their coordinated expression may represent a potential link between ERα-associated signaling and subtype-specific proliferative states. Our findings provide an exploratory framework for understanding transcriptional programs associated with ERα activity and breast cancer heterogeneity, supporting further investigation of this module, as a potential molecular tool for subtype characterization and discrimination.

## 2 Materials and methods

### 2.1 Study design and expression profiles

This study was designed as a retrospective, cross-sectional, observational analysis of BC transcriptomic data. BC samples were collected at Hospital San José, TecSalud (Monterrey, Mexico), during two time periods. The first period was between 2011 and 2014. It was approved by the Ethics and Research Committees of the Universidad Autónoma de Nuevo León, Hospital Universitario “José Eleuterio González” (Monterrey, Mexico) under project ID BI11-005. The second period occurred between 2017 and 2019, and this collection was approved by the Ethics and Research Committees of Tecnológico de Monterrey, Hospital San José, TecSalud (Monterrey, Mexico), under project ID P000088-Altru-Pro-CI-CR002.

Breast tumor biopsies (fresh-frozen tissue) representing all major BC subtypes were used for RNA isolation and global gene expression profiling using the GeneChip Human Genome U133 Plus 2.0 Array (Affymetrix; Thermo Fisher Scientific, Waltham, MA, USA). A total of 65 expression profiles derived from the breast tumor biopsies were analyzed, including 54 samples from the first collection and 11 from the second one. Corresponding clinicopathological data were compiled and incorporated into downstream analyses.

### 2.2 Molecular classification by PAM50

In addition to immunohistochemistry (IHC)-based subtyping (13), molecular classification of breast cancer samples was performed using the PAM50 gene expression signature (14). This method assigns intrinsic tumor subtypes based on the expression profiles of 50 classifier genes using a centroid-based approach.

Normalized microarray expression data from the 65 samples were used as input for PAM50 classification. The classifier was implemented in R following the centroid-based methodology described in the original PAM50 model. Gene matching between microarray probes and PAM50 genes was performed during preprocessing, and all classifier genes were represented in the dataset. Data normalization, centering, and scaling were handled within the PAM50 algorithm. Each sample was assigned to one of five intrinsic subtypes: LumA, LumB, HER2-enriched, Basal-like (corresponding to TN), and Normal-like. The resulting PAM50 subtype assignments were used to complement and refine IHC-based classification. Discrepancies between IHC and molecular subtypes were identified, and reclassified samples were incorporated into downstream analyses.

### 2.3 Principal component analysis on PAM50 subtypes for batch effect assessment

Furthermore, a Principal Component Analysis (PCA) was performed on TAC (Transcriptome Analysis Console) software (version 4.0.3.14) from Affymetrix (Thermo Fisher Scientific, Waltham, MA, USA) to visually assess sample clustering by dividing expression profiles according to collection period (2011–2014 or 2017-2019) and to determine whether samples exhibited clustering patterns suggestive of batch effects.

### 2.4 Literature and signaling pathways databases revision

Scientific journals from 2012 to 2022 were reviewed to identify genes associated (directly or indirectly) with ERα signaling. To identify peer-reviewed studies, a structured literature review was performed using PubMed (15) and Scopus (16) databases. To ensure broad coverage, Google Scholar (17) was used as a supplementary tool to identify additional relevant publications not captured in indexed databases.

Furthermore, genes were searched in databases and platforms for signaling pathways, including the Kyoto Encyclopedia of Genes and Genomes (KEGG, https://www.genome.jp/kegg/) (18), Wiki pathways (https://wikipathways.org) (19), Reactome Knowledgebase (https://reactome.org) (20), and the Ingenuity Pathway Analysis (IPA) software (QIAGEN Inc., https://digitalinsights.qiagen.com/citation-guidelines/) (21). A database was compiled from the selected genes from both searches. Shared genes were included only once, and duplicates, by symbol/gene name or synonym, were discarded.

### 2.5 Identification of differentially expressed genes

A supervised analysis was conducted using the TAC software (version 4.0.3.14) from Affymetrix. The analysis involved 65 expression profiles from BC patients and genes encoding proteins with reported nuclear localization that are fully or potentially associated with ERα. Subcellular localizations were obtained from UniProtKB/Swiss-Prot and The Human Protein Atlas databases. Samples were divided by molecular subtype (condition) and normalized and summarized using the RMA (Robust Multi-Array Average) algorithm (22). Resulting *p*-values were adjusted for multiple testing using the Benjamini–Hochberg false discovery rate (FDR) method (23). Genes with an FDR-adjusted *p*-value <0.0001 were considered significantly differentially expressed. For visualization, supervised hierarchical clustering was performed on genes with fold changes ≥ 2 or ≤ −2 and *p*-value ≤ 0.05.

### 2.6 Functional enrichment analyses

Identified differentially expressed genes (DEGs) were subjected to functional enrichment analyses, including Gene Ontology (GO) Biological processes analysis, transcription factor enrichment using ChIP-based datasets, and protein-protein interaction network analysis using STRING (24). The module was divided into gene sets based on their functions and expression patterns as inferred from the heatmaps.

### 2.7 Expression analysis of candidate genes in BC cell lines

#### 2.7.1 Gene selection

A subset of three DEGs with reported potential associations with ERα-related regulatory mechanisms in the literature but not fully characterized in curated signaling pathways, was selected from the nuclear module for further exploration. Expression of selected candidate genes was evaluated through a RT-qPCR in breast cancer cell lines. Gene selection was based on biological relevance and representation of expression patterns across PAM50 subtypes.

#### 2.7.2 Cell culture and reagents

Four different BC cell lines were obtained from the American Type Culture Collection (ATCC) and cultured for this project: MCF7 (LumA, code HTB-22), HCC1428 (LumB, code CRL-2327), HCC1954 (HER2+, code CRL-2338), and MDA-MB-231 (TN, code HTB-26). MCF7 and MDA-MB-231 were grown in Dulbecco’s Modified Eagle Medium (DMEM, 1X) medium (GIBCO, Thermo Fisher Scientific, Waltham, MA, USA) (catalog number 11995065). HCC1428 and HCC1954 were cultured in Roswell Park Memorial Institute (RPMI-1640, 1X) medium (American Type Culture Collection [ATCC], Manassas, VA, USA) (catalog number 30-2001). Growth mediums (aliquots) were supplemented with 10% qualified fetal bovine serum/FBS (GIBCO, Thermo Fisher Scientific, Waltham, MA, USA) (catalog number 26140079) and 1% Antibiotic-antimycotic/AA 100X (GIBCO, Thermo Fisher Scientific, Waltham, MA, USA) (catalog number 15240-062). The four cell lines were maintained in a CO_2_ incubator model MCO-18AIC (SANYO Electric Biomedical Co., Ltd., now Panasonic, Kadoma, Japan) at 5% CO2 and a constant temperature of 37 °C.

All cell lines were initially cultured in a 25 cm^2^ cell culture flask, with the medium renewed every two to three days. When the flasks reached the 80-90% confluency, cells were detached by incubation with two to three milliliters of 0.25 trypsin-EDTA 0.25% (GIBCO, Thermo Fisher Scientific, Waltham, MA, USA) (catalog number 25200072). Detached cells were then counted with a 1:1 dilution of medium with trypan blue stain 0.4% (GIBCO, Thermo Fisher Scientific, Waltham, MA, USA) (catalog number, 15250061) in a Neubauer chamber (Paul Marienfeld GmbH & Co. KG, Lauda-Königshofen, BW, Germany) (catalog 0610010). After counting, cells were subcultured into a 75 cm^2^ cell culture flask and maintained until they reached 80-90% confluency again. The time to reach high confluency varied among the four cell lines. Once confluent, cells were detached and counted again. For most cell lines, 3 million cells (1 million for HCC1954) were placed in a microtube and frozen at -20 °C until RNA isolation. Procedures for cell cryopreservation, thawing, and subculture were performed in accordance with the ATCC Animal Cell Culture Guide (https://www.atcc.org/resources/culture-guides/animal-cell-culture-guide).

#### 2.7.3 RNA isolation and RT-qPCR

From three (MCF7, HCC1428, MDA-MB-231) or one (HCC1954) million cells, total RNA was extracted using the RNeasy Mini kit protocol (QIAGEN N.V., Germantown, MD, USA). RNA concentration was determined by NanoDrop 2000 spectrophotometer (Thermo Fisher Scientific, Waltham, MA, USA). cDNA was then generated with the High-Capacity cDNA Reverse Transcription kit (Thermo Fisher Scientific, Waltham, MA, USA), and this program: 25 °C – 10 min, 37 °C – 120 min, 85 °C – 5 min, and 4 °C – Hold, for enzyme activation in the Veriti 96-well Thermal Cycler (Thermo Fisher Scientific, Waltham, MA, USA). Subsequently, the qPCR (Quantitative Polymerase Chain Reaction) was performed with the cDNA, the TaqMan Fast advanced master mix (Thermo Fisher Scientific, Waltham, MA, USA), and the TaqMan Gene Expression Assay probes (Thermo Fisher Scientific, Waltham, MA, USA): E2F2 - Hs00918090_m1, PARP1 - Hs00242302_m1, PAK2 - Hs02559219_s1, and GAPDH - Hs03929097_g1.

qPCR parameters were the following: 95 °C – 20 sec for 1 cycle, and 40 cycles of 95 °C – 1 sec (denaturation) as well as 60 °C – 20 sec (annealing) in the QuantStudio 3 Real-Time PCR Instrument (Thermo Fisher Scientific, Waltham, MA, USA). Delta (Δ) Ct values were compared to obtain relative expression of each of the three genes (*E2F2, PARP1*, and *PAK2*), in reference to the housekeeping gene *GAPDH*. **Supplementary Figure 1** (**Supplementary Material 1**) presents a graph with corresponding GAPDH Ct values.

### 2.8 *In silico* expression analysis of the 34-gene module in an independent BC cohort

Analysis of the nuclear module expression reproducibility was conducted in SurvExpress (http://victortrevino.bioinformatics.mx:8080/Biomatec/SurvivaX.jsp), an online biomarker validation platform and database for cancer gene expression data (25), using GSE18229 public BC cohort (26). This database was selected because its samples were classified according to PAM50 subtypes, consistent with the molecular classification applied to our cohort. The analyzed cohort (N = 199) consisted of expression profiles derived from 182 breast tumors and 17 normal breast tissues (primarily reduction mammoplasties). Expression profiles of the 34 genes in the nuclear module were analyzed using hierarchical clustering (*p* < 0.05).

### 2.9 Gene expression correlation analysis of the 34-gene module versus *MKI67*

Statistical analyses and data visualization were performed to identify correlation patterns among the 34-gene module transcripts and Ki-67/*MKI67* expression. R software was employed for this analysis (RStudio 2023.03.0, www.R-project.org/) (27, 28). Pearson correlation coefficients (*r)* and their corresponding p-values were calculated to evaluate linear associations between the expression levels of the evaluated genes. The threshold for statistical significance was set at *p* < 0.05. For heatmap construction, cells were colored using a combined metric: the direction of the correlation (positive or negative) and the strength of its statistical significance (-\log_10 of the p-value). Associations that failed to reach statistical significance (*p* > 0.05) were visually masked by assigning them a value of zero, thereby highlighting only biologically robust interactions.

### 2.10 Comparative analysis of the 34-gene module and BC genomic signatures

A comparative analysis was performed between the proposed 34-gene module and four established BC genomic signatures: EndoPredict, Oncotype DX, Prosigna (PAM50) and MammaPrint. The comparison included associated biological processes, clinical utility, target patient population, strengths, limitations, and genes shared with our signature, based on information obtained from the original publications and current clinical literature.

### 2.11 Statistical analysis

Descriptive statistics and measures of central tendency were used to assess associations between each BC molecular subtype group and the clinicopathological variables. Differences between the comparison groups were analyzed using Student’s T-tests for quantitative variables and *Chi*-squared tests, or, failing that, Fisher’s exact test for qualitative variables. Regarding the qPCR, ΔCt values were compared using ANOVAs to determine the BC cell line with the highest abundance of each gene. The P-values for the ANOVAs were <0.0001. Statistical analyses for experimental evaluation were executed using GraphPad Prism software (GraphPad Software Inc., San Diego, CA).

## 3 Results

### 3.1 PAM50 vs IHC

BC samples were reclassified using the PAM50 algorithm to achieve more accurate and uniform stratification of BC (**Supplementary Table 1**, **Supplementary Material 1**). Initially, the IHC subtype distribution corresponded to 12 LumA, 7 LumB, 7 HER2+, and 38 TN samples. After reclassification using PAM50, samples were stratified as follows: 23 LumA, 8 LumB, 5 HER2-enriched, 25 basal, and 4 normal-like. Of the 65 samples, 25 (38%) changed subtype. The subtype with the highest level of reclassification (IHC to PAM50) was HER2+ (5/7 = 71%), followed by LumB (3/7 = 43%), TN (13/38 = 34%), and LumA (3/12 = 25%). Only one of the 65 samples lacked subtype by IHC.

HER2+ samples (by IHC) were mostly changed to LumA (4/5 = 80%); all the reclassified LumB samples were assigned to LumA (3/3 = 100%); the TN samples were mainly reclassified as LumA (7/13 = 54%), and most LumA samples reclassified changed to LumB (2/3 = 67%). The IHC-undetermined subtype corresponded to LumB by the PAM50 method.

Subtype distribution across the two sample collection periods (2011–2014 and 2017-2019) is shown in **Supplementary Table 2** (**Supplementary Material 1**). Furthermore, clinicopathological characteristics of the 65 BC patients included in this study are summarized in **Table 1**.

**Table 1:** Clinicopathological characteristics of 65 BC patients whose expression profiles were used in this study (2011-2014 and 2017-2019).

Characteristics	Subtypes by IHC										
	LumA		Lum B		HER2+		TN		Undescribed sample		p-value
	n=12	(%)	n=7	(%)	n=7	(%)	n=38	(%)	n=1	(%)	
Age											0.6587^&^
	(Avg 46 y)		(Avg 51 y)		(Avg 48 y)		(Avg 50 y)		(39 y)		
≤40 years	3	25.0	0	0.0	2	28.6	6	15.8	1	100.0	
>40 years	9	75.0	7	100.0	5	71.4	32	84.2	NA	NA	
Menopausal status											0.6174^#^
Premenopausal	6	50.0	4	57.1	2	28.6	21	55.3	NA	NA	
Postmenopausal	6	50.0	3	42.9	5	71.4	17	44.7	1	100.0	
BMI											0.7829^&^
	(Avg 28)		(Avg 30)		(Avg 30)		(Avg 28)		N/A		
20.0-24.9 (healthy)	2	16.7	2	28.6	1	14.3	6	15.8	NA	NA	
25.0-29.9 (overweight)	6	50.0	1	14.3	2	28.6	17	44.7	NA	NA	
30.0-39.9 (obese)	4	33.3	3	42.9	3	42.9	9	23.7	NA	NA	
≥40.0 (morbid obese)	0	0.0	1	14.3	0	0.0	1	2.63	NA	NA	
NS	0	0.0	0	0.0	1	14.3	5	13.2	1	100.0	
Cancer clinical stage											0.7082^#^
IA	0	0.0	0	0.0	1	14.3	0	0.0	NA	NA	
IIA y IIB	6	50.0	3	42.9	3	42.9	14	36.9	1	100.0	
IIIA, IIIB y IIIC	6	50.0	4	57.1	3	42.9	24	63.2	NA	NA	
Family history of cancer											0.2151^#^
Yes	7	58.3	3	42.9	1	14.3	21	55.3	NA	NA	
No	5	41.7	4	57.1	6	85.7	17	44.7	1	100.0	
Overall recurrence (5 years)											0.4255^#^
Yes	3	25.0	2	28.6	0	0.0	11	28.9	NA	NA	
No	9	75.0	5	71.4	7	100.0	26	68.4	1	100.0	
NS	0	0.0	0	0.0	0	0.0	1	2.63	NA	NA	
	Subtypes by PAM50										
Characteristics	LumA		LumB		HER2-enriched		Basal		Normal-like		p-value
	n=23	(%)	n=8	(%)	n=5	(%)	n=25	(%)	n=4	(%)	
Age											0.4761^&^
	(Avg 50 y)		(Avg 53 y)		(Avg 53 y)		(Avg 47 y)		(Avg 45 y)		
≤40 years	4	17.4	1	12.5	0	0.0	6	24.0	1	25.0	
>40 years	19	82.6	7	87.5	5	100.0	19	76.0	3	75.0	
Menopausal status											0.0237^*#^
Premenopausal	11	47.8	2	25.0	0	0.0	17	68.0	3	75.0	
Postmenopausal	12	52.2	6	75.0	5	100.0	8	32.0	1	25.0	
BMI											0.9445^&^
	(Avg 29)		(Avg 28)		(Avg 32)		(Avg 28)		(Avg 29)		
20.0-24.9 (healthy)	3	13.0	2	25.0	0	0.0	5	20.0	1	25.0	
25.0-29.9 (overweight)	11	47.8	1	12.5	1	20.0	12	48.0	1	25.0	
30.0-39.9 (obese)	7	30.4	3	37.5	3	60.0	4	16.0	2	50.0	
≥40.0 (morbid obese)	1	4.3	0	0.0	0	0.0	1	4.0	0	0.0	
NS	0	0.0	2	25.0	1	20.0	3	12.0	0	0.0	
Cancer clinical stage											0.2416^#^
IA	1	4.3	0	0.0	0	0.0	0	0.0	0	0.0	
IIA y IIB	11	47.8	5	62.5	1	20.0	7	28.0	3	75.0	
IIIA, IIIB y IIIC	11	47.8	3	37.5	4	80.0	18	72.0	1	25.0	
Family history of cancer											0.4663^#^
Yes	10	43.5	2	25.0	3	60.0	15	60.0	2	50.0	
No	13	56.5	6	75.0	2	40.0	10	40.0	2	50.0	
Overall recurrence (5 years)											0.6036^#^
Yes	7	30.4	1	12.5	1	20.0	7	28.0	0	0.0	
No	15	65.2	7	87.5	4	80.0	18	72.0	4	100.0	
NS	1	4.3	0	0.0	0	0.0	0	0.0	0	0.0	

*Significant value, p<0.05. P-value according to Chi-squared 11.27.

Avg, Average; BMI, Body mass index; IHC, Immunohistochemistry; NA, Not applicable; NS, Not shown.

^&^ANOVA test.

^#^Chi-squared test.

### 3.2 Principal component analysis for batch effect assessment

In addition, PCA (**Supplementary Figure 2**, **Supplementary Material 1**) was performed to identify potential batch-associated clustering patterns among the PAM50 subtypes. PCA revealed substantial overlap between samples collected during 2011–2014 and 2017-2019, indicating no evident global batch effect associated with the collection period. Although three samples from the 2011–2014 cohort (S_08 – Basal-like, S_27 – LumA, and S_161–Normal-like) appeared separated from the main cluster (possibly reflecting biological or sample-specific variation rather than systematic effect), the remaining samples from both periods showed a high degree of intermixing, suggesting that temporal batch effects were not a major source of variation in the dataset. In addition, only one of the two plots displayed in **Supplementary Material 1** was shaped by PAM50-subtypes; however, PAM50 annotation did not alter the overall sample distribution, indicating that the observed clustering was not solely driven by intrinsic subtype classification (29).

### 3.3 Associations between clinicopathological characteristics and BC subtypes

Several clinicopathological characteristics were evaluated according to the IHC and PAM50-based subtypes (**Table 1**). Among the 65 BC patients, age, menopausal status, BMI, clinical stage, family history of cancer, and overall recurrence were assessed. No statistically significant associations were identified between the clinical parameters and the IHC subtypes. On the other hand, a significant association was observed between the PAM50 molecular subtypes and menopausal status (*p-value = 0.0237*). Nevertheless, given the unequal distribution of PAM50 subgroups within our cohort and sample sizes ranging from 4 to 25, these results should be interpreted with caution.

### 3.4 ERα-related genes database

#### 3.4.1 Literature and signaling platforms revision

**Figure 1:**
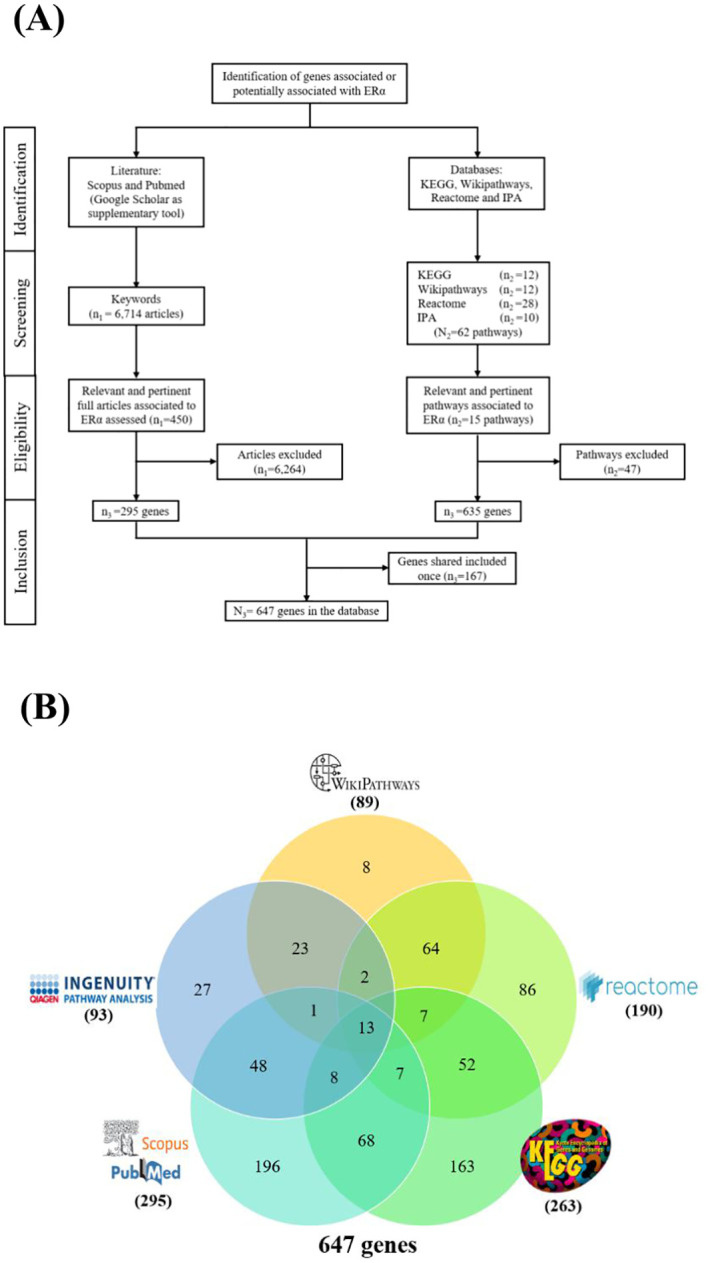
**(A, B)** Workflows of identification of genes associated or potentially associated with the ERα signaling pathway. **(A)** Structured diagram of identified, screened, eligible, and included articles (n_1_), pathways (n_2_), and genes (N_3_) for this study. Articles were retrieved from Scopus and PubMed. A secondary source (Google Scholar) was used as a complement. The search terms included “Estrogen receptor alpha” OR “ERα” OR “ESR1” AND “signaling pathway” OR “signaling cascade” AND “regulates” OR “modulates” OR “regulator” OR “modulator” AND “associated” OR “associates” AND “target gene”. **(B)** Venn diagram representation of the shared genes amongst all the sources of the 647 genes found as associated with the ERα. The signaling database that shared the most genes with the literature was KEGG (68 genes), and the one with the fewest genes in common was Wikipathways (30 genes). The platforms with the highest number of shared genes were Reactome and Wikipathways (64 genes).

Genes reported in the literature were included in the database if evidence of functional or regulatory associations with ERα signaling was provided (**Figure 1A**). Priority was given to experimentally validated interactions. For instance, Lu and colleagues (30) published a survival analysis showing elevated *Forkhead Box M1* (*FOXM1*) expression and predicting poor clinical outcome and early recurrence (HR = 1.85, p = 5.8e–10) in hormone-positive BC tumors. This can be interpreted as a possible association of FOXM1 with the ERα signaling pathway. Furthermore, Zhou et al. (31) reported that the *S-phase kinase associated protein 2* (*SKP2*) coactivates, along with the ERα, G1/S transition genes in MCF7 ER-positive cells. Wierer and his team (32) validated a direct interaction of the *Polo-Like Kinase 1 (PLK1)* with the ERα, by co-regulating transcription of ER-target and cell cycle-related genes in MCF7 cells.

Other examples are *SMARCA4* and *EZH2*. Jeong *et al.* (33), demonstrated that the *SWI/SNF related, matrix associated, actin dependent regulator of chromatin, subfamily a, member 4* (*SMARCA4, also called BRG1)* co-regulates, along ERα, the transcription of *Trefoil Factor1* (*TFF1)* in MCF7 cells. According to Wu *et al.* (34), the *enhancer of zeste 2 polycomb repressive complex 2 subunit* (*EZH2*) influences ERα transcriptional activity in tamoxifen-resistant cells, via the EZH2-ERα-GREB1 axis.

On the other hand, genes were also retrieved from curated signaling databases (KEGG, Reactome, Wikipathways, and IPA) (**Figure 1B**). Signaling pathways linked to ERα activity, such as estrogen signaling, estrogen receptor signaling, breast cancer, and endocrine resistance, were explored, and participant genes were added to our database. Out of the 647 genes compiled, thirteen genes are shared by all sources (literature + signaling databases): *AKT1, BCL2, CCND1, ESR1, ESR2, FOS, HSP90AA1, IGF1R, JUN, MMP2, PIK3CA, SHC1*, and *SP1*.

### 3.5 Differential expression analysis

#### 3.5.1 Differentially expressed genes

Based on subcellular localization annotations, 490 genes corresponded to proteins with reported nuclear localization, while 438 and 383 genes were annotated with cytoplasmic and membrane localization, respectively. Included proteins may have dual or multiple compartment annotations.

**Figure 2:**
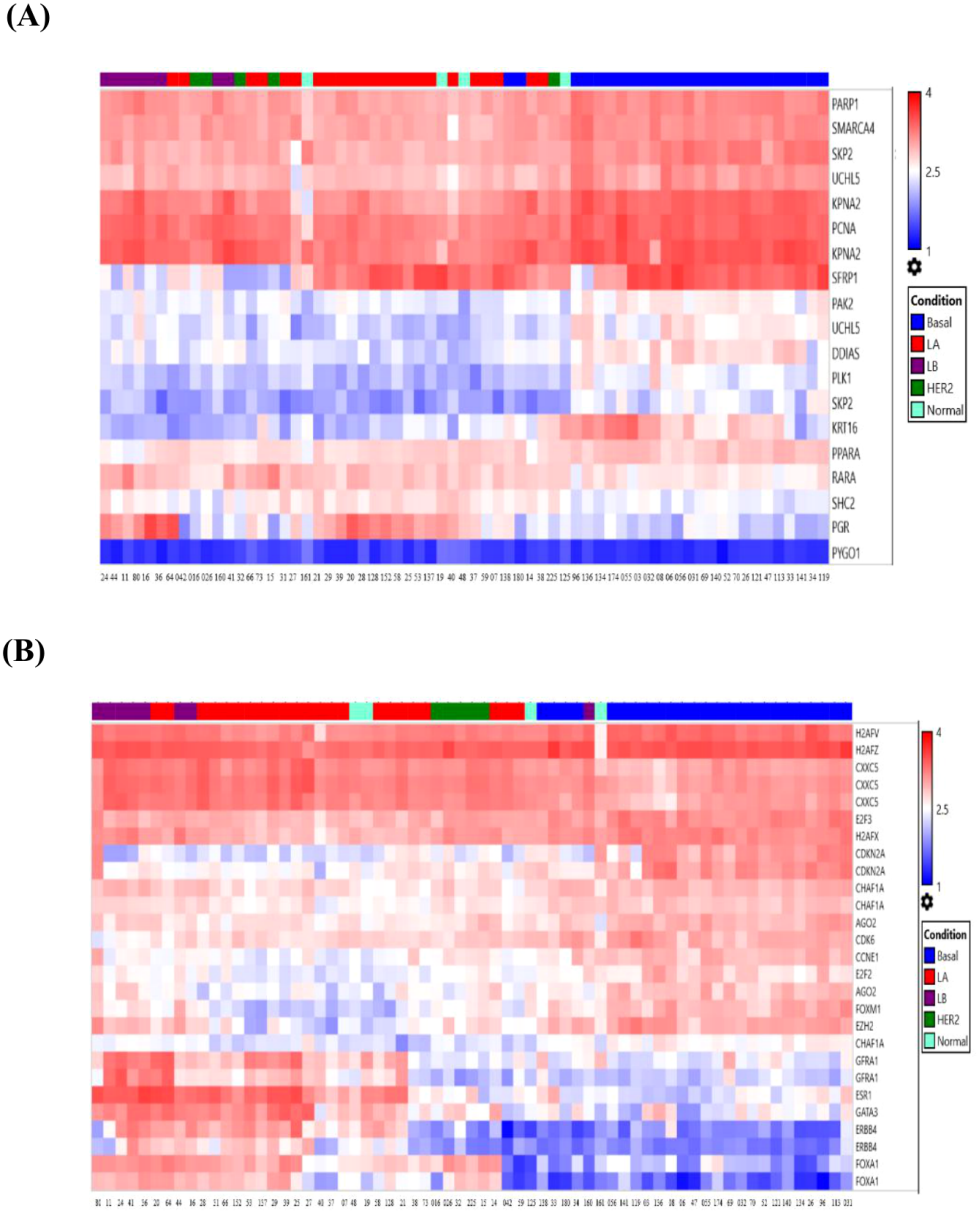
**(A, B)** Heatmaps generated from the supervised analysis, encompassing genes with nuclear-localized proteins (n=490) and the BC expression profiles (N = 65). **(A)** Heatmap of the first half of the 490 genes (n=245) with an FDR<0.0001. **(B)** Heatmap of the second half of the 490 genes (n=245) with an FDR<0.0001. Expression by subtype (condition). Blue cells represent low gene expression, and red corresponds to high gene expression. Each column is a patient, and each row is a gene. Plots obtained with TAC software (Affymetrix). LA: luminal A; LB: luminal B; HER2: HER2-enriched; Basal: Basal-like; Normal: Normal-like. Gene symbols in the heatmap correspond to the annotations provided by the Affymetrix TAC platform. According to the Hugo Gene Nomenclature Committee (HGNC), H2AFZ and H2AFV correspond to the current gene symbols H2AZ1 and H2AZ2, respectively.

The supervised analysis, using only nuclear protein genes and expression profiles, yielded two heatmaps. Initially, heatmaps included all analyzed genes and illustrated the global expression patterns across samples (**Supplementary Figure 3**, **Supplementary Material 1**). After the statistical filter, heatmaps (**Figures 2A, B**) showed the most significantly expressed genes after multiple testing correction, with fold changes >2 or <- 2 and FDR <0.0001. Genes that appear more than once correspond to multiple probe sets. In total, 34 DEGs are shown: *AGO2, CCNE1, CDK6, CDKN2A, CHAF1A, CXXC5, DDIAS, E2F2, E2F3, ERBB4, ESR1, EZH2, FOXA1, FOXM1, GATA3, GFRA1, H2AFX, H2AZ1* (*H2AFZ* on heatmap), *H2AZ2* (*H2AFV* on heatmap*), KPNA2, KRT16, PAK2, PARP1, PCNA, PGR, PLK1, PPARA, PYGO1, RARA, SFRP1, SHC2, SKP2, SMARCA4*, and *UCHL5*.

#### 3.5.2 Gene expression patterns in BC subtypes

Heatmaps depict a gene panel differentially expressed across BC molecular subtypes, showing: A. general upregulation *(PARP1, SMARCA4, SKP2, UCHL5, KPNA2, PCNA, SFRP1, H2AZ1, H2AZ2, CXXC5, E2F3, H2AFX, CDKN2A, CHAF1A, AGO2, CDK6, PPARA* and *RARA*; B. genes showing expression patterns enriched in one or more proliferative subtype (LumB, HER2-enriched and basal) and downregulation in non-proliferative tumors (*CCNE1, E2F2, AGO2, FOXM1, EZH2, CHAF1A, PAK2, UCHL5, DDIAS, PLK1, SKP2* and *KRT16*); C. upregulation in LumA, LumB and normal-like with variable expression patterns in HER2-enriched and basal (*GFRA1, ESR1, GATA3, ERBB4, FOXA1, SHC2* and *PGR*) and finally D. general downregulation (*PYGO1*). **Supplementary Table 3** (**Supplementary Material 1**) compiles the gene set from the heatmaps, along with their fold changes>2 or <-2, FDR values <0.0001, and their established or potential association with ERα.

#### 3.5.3 Functional enrichment and network analyses

**Figure 3:**
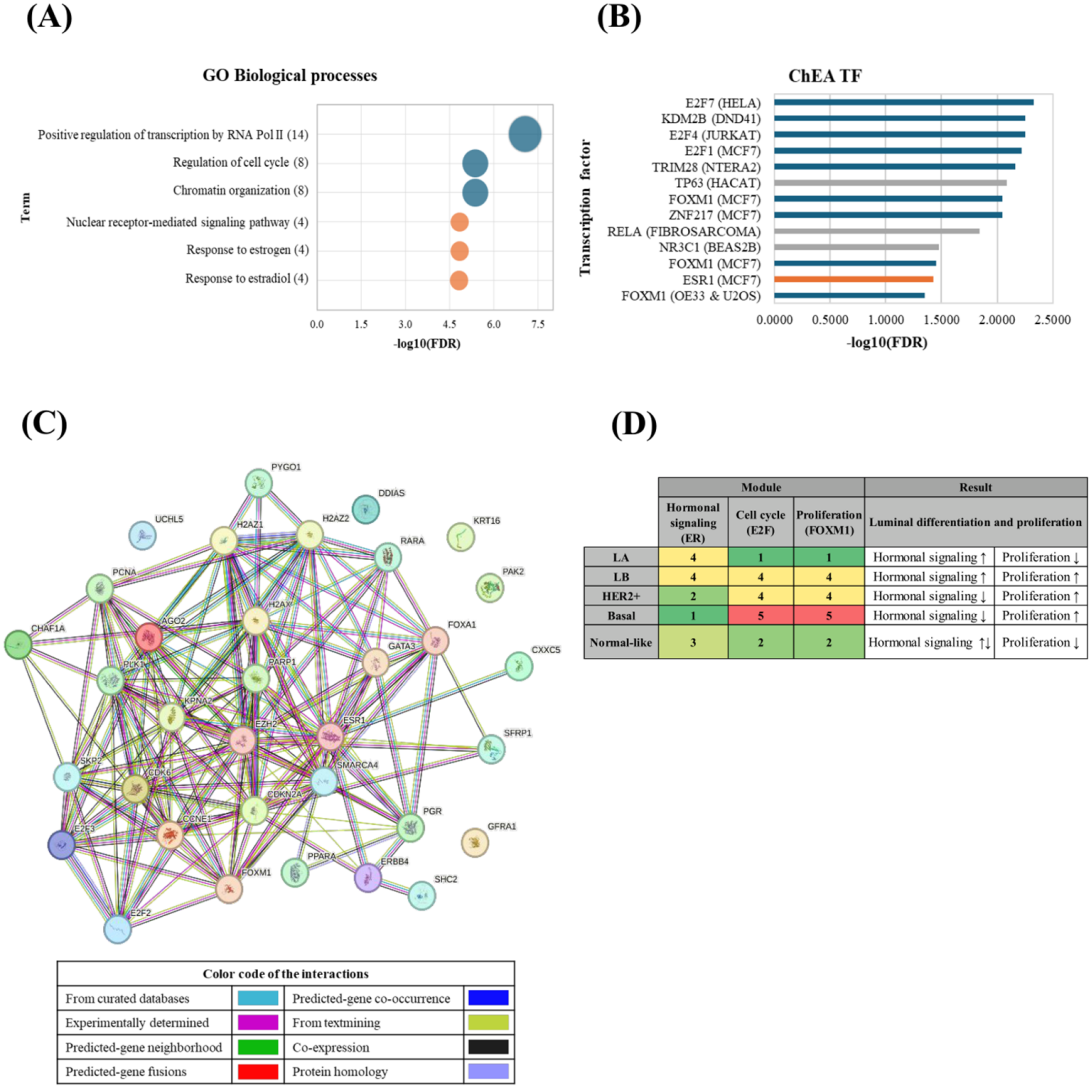
**(A–D)** Enrichment analyses of the 34 DEGs and identification of the ERα-E2F/FOXM1 axis. **(A)** Bubble plot representing the enriched GO biological processes for the 34-gene nuclear module. Selected enriched GO terms relevant to nuclear receptor signaling, transcription, and cell cycle regulation from the top 15 are shown. The x-axis shows −log10(FDR), and bubble size reflects the number of genes associated with each term from the y-axis. Blue bubbles represent genes associated with transcription/cell cycle regulation processes, and the orange bubbles indicate genes involved in estrogen signaling processes. Adjusted p-value <0.05. GO analysis performed on Enrichr. **(B)** Bar plot of the top 13 transcription factors identified in the ChEA from Enrichr. The x-axis shows −log10(FDR), while the y-axis describes transcription factors and human cellular lines (in brackets) as their source. Blue bars represent transcription factors whose target genes in our module are associated with transcription/cell cycle/chromatin regulation; gray bars represent genes not considered for our module; and the orange bar represents the gene linked to estrogen signaling. Adjusted p-value <0.05. **(C)** STRING protein-protein interaction network of the 34-gene nuclear module. Interaction confidence score ≥0.4. Circles (nodes) represent genes. Interactions among genes are shown as lines of different colors. The color code is described in the table below. **(D)** Categorical heatmap of the potential features of the ERα-E2F/FOXM1 axis across the BC subtypes. LA, luminal A and LB, luminal B. 1: low (moderate green), 2: low-moderate (light green), 3: moderate (yellow-green), 4: high (yellow), and 5: very high (red). Arrows represent high (↑) and low (↓) levels. Both arrows (↑↓) represent a moderate result.

**Figures 3A, B** represents the functional enrichment analyses executed on Enrichr from the Ma’ayan laboratory (35). The top 15 enriched biological processes (**Supplementary Table 4**, **Supplementary Material 1**) of the gene ontology analysis (probability of any gene belonging to any of the processes, *p*-value<0.05) showed two types of processes; i) transcription, chromatin and cell cycle regulation (blue bubbles) with 14 genes involved in transcription, 8 in cell cycle and another 8 in chromatin regulation; while ii) hormonal signaling (orange bubbles) was linked to 4 genes for nuclear receptor pathway, another 4 in response to estrogen and 4 more in response to estradiol (**Figure 3A**). Transcription factor enrichment analysis using ChEA (**Figure 3B**) revealed significant enrichment for targets of E2F family members (*E2F7, E2F4, and E2F1*) and FOXM1, as well as *KDM2B, TRIM28*, and *ZNF217*, known regulators of cell cycle progression and chromatin regulation, respectively. Additionally, targets of *ESR1* (orange bar) were also notably enriched (adjusted *p* = 0.029), supporting the involvement of ERα signaling in this nuclear gene module. Other significant transcription factors identified were TP63, RELA, and NR3C1 (gray bars), which have been linked to inflammatory signaling.

Protein–protein interaction network analysis using STRING (**Figure 3C**) exposed a highly connected network among the 34 nuclear genes with a PPI (protein-protein interaction) enrichment p-value <1.0×10⁻¹^6^. Combined scores for the 166 interactions in the gene module are shown in **Supplementary Table 5** (**Supplementary Material 1**). The most interacting genes were *CDKN2A* (18 interactions), *ESR1* 16 interactions, and *CCNE1* as well as *EZH2*.

#### 3.5.4 Functional roles in BC subtypes

DEGs (N = 34) were organized into three functional modules or groups based on their reported biological roles in BC, including both direct and indirect contributions to relevant cellular processes.

Group 1. Hormonal signaling (n=11): *ESR1, PGR, FOXA1, GATA3, ERBB4, GFRA1, SFRP1, PPARA, RARA, CXXC5* and *SHC2*; it includes genes highly associated with the ERα signaling. Group 2. Proliferative/cell cycle (n=10): *E2F2, E2F3, FOXM1, CCNE1, CDK6, CDKN2A, SKP2, PLK1, EZH2*, and *KPNA2*; these genes can influence cell division and mitogenic features. Group 3. Genome regulation and cancer-related processes (n=13): *PCNA, PARP1, H2AFX, H2AZ1, H2AZ2, SMARCA4, CHAF1A, AGO2, UCHL5, PAK2 DDIAS* and *KRT1*6. This set serves as a modulator (activators or repressors, e.g., *SMARCA4* and *UCHL*5) of the genome and includes cancer-related processes as well.

#### 3.5.5 Expression vs function

Groups 1, 2, and 3 were compared with groups A, B, C, and D. Genes with general upregulation across subtypes (group A) coincided with some of those associated with the genome regulation and cancer-related processes from group 3 (*PARP1*, *SMARCA4, PCNA, CHAF1A, H2AFX, H2AZ1, H2AZ2* & *UCHL*5). Other genes associated with hormonal group (group 1) coincided with increased expression of hormonal and normal-like subtypes and downregulation of proliferative samples from group C (*GFRA1, ESR1, GATA3, ERBB4, FOXA1, SHC2* and *PGR*); moreover, genes associated with proliferation/cell cycle regulation (group 2) corresponded to the group showing expression patterns enriched in one or more proliferative subtype (LumB, HER2-enriched and basal) and downregulation in most LumA and normal-like samples, group B (*CCNE1, E2F2, FOXM1, PLK1* & *SKP2*).

### 3.6 Identification of a nuclear program

#### 3.6.1 Coordinated nuclear module

Functional enrichment analyses and the protein-protein interaction analysis allowed the identification of a nuclear transcriptional module. *AGO2, CCNE1, CDK6, CDKN2A, CHAF1A, CXXC5, DDIAS, E2F2, E2F3, ERBB4, ESR1, EZH2, FOXA1, FOXM1, GATA3, GFRA1, H2AFX, H2AFZ*, *H2AFV, KPNA2, KRT16, PAK2, PARP1, PCNA, PGR, PLK1, PPARA, PYGO1, RARA, SFRP1, SHC2, SKP2, SMARCA4*, and *UCHL5* were recognized as a highly interacting circuitry. This module integrates expression patterns and the corresponding functions associated with the BC subtypes, showing biological coherence.

#### 3.6.2 E2F/FOXM1 has a potential regulatory role in the module

The BC nuclear gene module suggests the involvement of ESR1 (hormonal signaling), E2F family members (cell cycle regulation), and FOXM1 (proliferation) in synergy. The ERα-E2F/FOXM1 axis could be differentially regulated across BC subtypes. **Figure 3D** summarizes the proposed potential participation of the axis according to the biological background of each subtype. LumA and normal-like subtypes could display high and moderate (respectively) abundance of hormonal signaling (ERα activity) and low levels of proliferation genes (E2F family and FOXM1), contrary to LumB, HER2-enriched and basal groups, whose tumors are known for their aggressiveness and high proliferation.

#### 3.6.3 Connection of E2F/FOXM1 to the estrogen receptor alpha

Results from the analysis of GO biological processes showed enrichment for ERα-related activities, including the nuclear receptor-mediated signaling pathway, estrogen signaling, and estradiol signaling. Moreover, *ESR1* appeared as an enriched transcription factor (ChIP database), whose targets can be found in the module (*PGR, FOXA1, GATA3, ERBB4, SFRP1, RARA* and *CXXC5*). ERα is a known transcriptional regulator of cell cycle progression-related genes. **Supplementary Table 5** displays protein-protein interactions (score) of *ESR1* with SKP2 (0.554), PLK1 (0.42) and FOXM1 (0.79), all targets of the E2F transcription factor family. ERα-E2F/FOXM1 axis may contribute to the transcriptional regulation of the module.

### 3.7 Expression analysis of candidate genes in BC cell lines

#### 3.7.1 Gene selection

**Figure 4:**
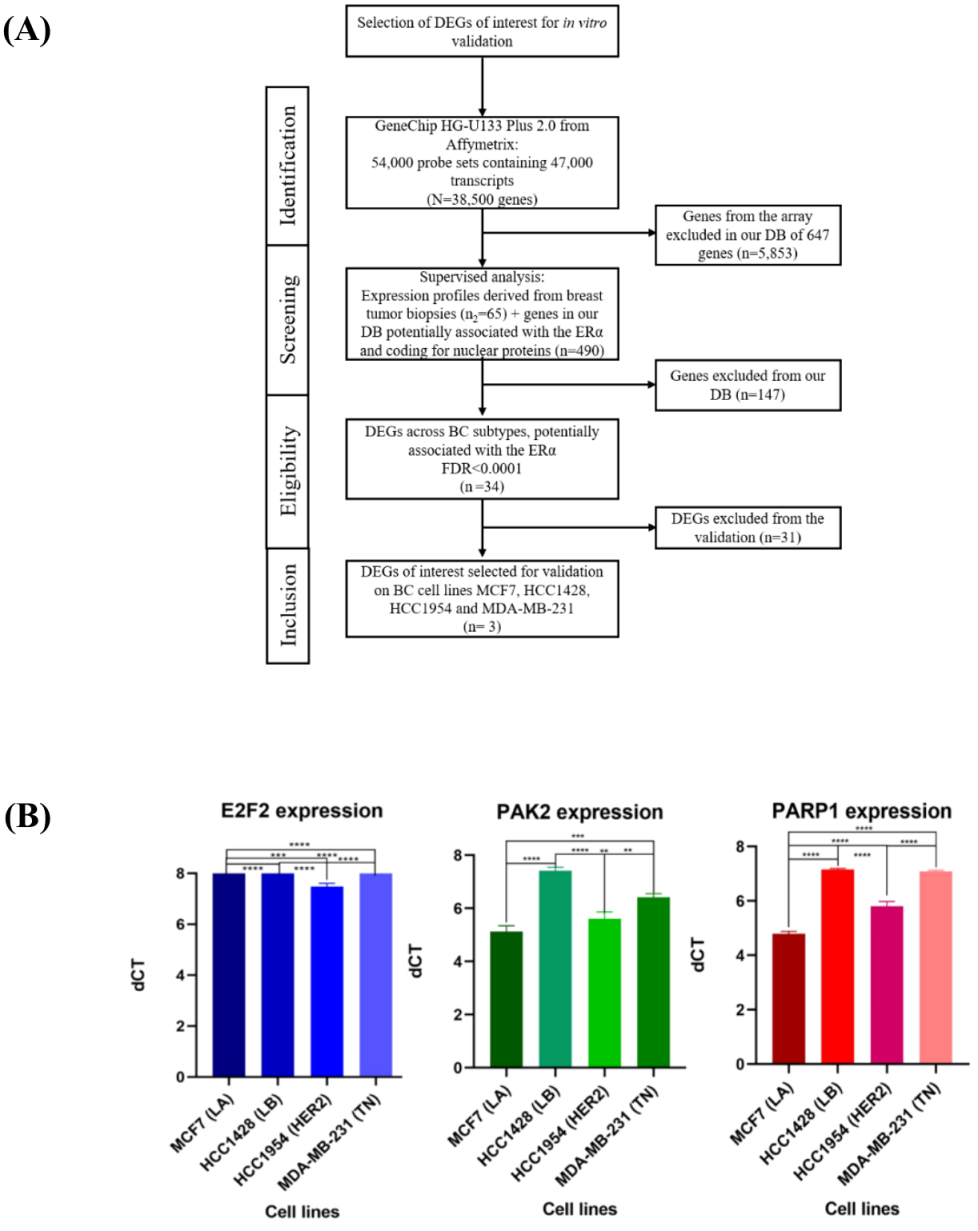
**(A, B)** Selection and expression evaluation of three DEGs out of the nuclear module. **(A)** Structured diagram of identified, screened, eligible, and included genes from the HG-U133 Plus 2.0 array (Affymetrix). **(B)** Bar plot showing *E2F2, PARP1*, and *PAK2* expression levels in human BC cell lines. Each bar represents the relative expression (Δ Ct value, Y axis) of a gene for a certain BC cell line (X axis). A lower Δ Ct value indicates a higher level of target gene expression. Orange arrows indicate the cell line with the highest abundance of *E2F2, PARP1*, or *PAK2*. P-value <0.001. *= p<0.05, **= p<0.01, *= p<0.001, **= p<0.0001, ***= p<0.00001.

Out of the 34 DEGs, three genes, E2F transcription factor 2 (*E2F2*), Poly (ADP-ribose) polymerase 1 (*PARP1*), and P21-activated kinase 2 (*PAK2*), were selected for further experimental evaluation (**Figure 4A**). Based on the integration of expression patterns and functional categorization, *E2F2* corresponded to groups 2 and B and was selected as a representative of the E2F-related proliferative transcriptional program. *PARP1* was associated with groups 3 and A, corresponding to genome regulation and cancer-related processes and a broader expression pattern across PAM50 subtypes. *PAK2* was also included within group 3 and showed an expression pattern associated with group B, characterized by enrichment in proliferative subtypes (LumB, HER2-enriched, and TN) and relatively low expression patterns associated with luminal and normal-like subtypes. *PAK2* and *PARP1* were selected as representative genes associated with transcriptional regulation-related components of the module.

#### 3.7.2 Expression of *E2F2, PARP1* and *PAK2* in BC cell lines

Statistical comparisons (p<0.0001) of the RT-qPCR data (**Figure 4B**) show that when contrasting each gene expression (in reference to *GAPDH*) amongst all the BC cell lines, *E2F2* is enriched in HCC1954 (HER2+, ΔCt 7.48), while *PARP1* (ΔCt 4.79) and *PAK2* (ΔCt 5.13) are increased in MCF7 (LumA) (p<0.0001).

### 3.8 *In silico* expression analysis of the 34-gene module in an independent BC cohort

Within the expression reproducibility analysis in the Prat, Perou, and colleague’s cohort, three of the ERα targets, *SFRP1, PGR, and FOXA1* emerged as representative genes of the nuclear module (**Supplementary Figure 4**, **Supplementary Material 1**), displaying in the heatmap subtype-associated expression patterns consistent with those observed in our original dataset. *SFRP1* showed overexpressed patterns in LumA, normal and basal subtypes; whereas *FOXA1* was overexpressed in LumA, LumB and HER2-enriched, and *PGR* showed evident overexpression in LumA and LumB samples in comparison with proliferative subtypes. Notably, these genes showed differential expression across PAM50 subtypes, recapitulating the transcriptional trends identified in our cohort.

### 3.9 Global analysis of the gene co-expression matrix

Bivariate Pearson correlation analysis revealed a heterogeneous and compartmentalized co-expression pattern among the evaluated transcripts. Conditional mapping within the heatmap allowed for immediate discrimination of statistically robust associations from background noise, as evidenced by biologically non-significant regions (*p >*0.05) that remained neutral in white. The global landscape displays blocks of coordinated co-expression, suggesting the existence of interconnected functional networks within the analyzed dataset.

#### 3.9.1 Directed correlation profile: 34-gene module versus *MKI67*

A directed analysis of the first column of the matrix (**Supplementary Figure 5**, **Supplementary Material 1**), which evaluates the transcriptional behavior of the 34 genes against MKI67 (a molecular marker of cell proliferation), identified the most relevant and statistically potent transcriptional associations in the study (**Supplementary Table 6**, **Supplementary Material 2**): High-magnitude positive associations (red saturation): A cluster of genes strongly co-expressed with the proliferation signature was identified, led by *EZH2* (*r* = 0.8276, 95% CI: [0.7313 to 0.8915], *p* < 0.0001), *CHAF1A* (*r* = 0.8202, 95% CI: [0.7205 to 0.8867], *p* < 0.0001), *CCNE1* (*r* = 0.8156, 95% CI: [0.7137 to 0.8837], *p* < 0.0001) and *H2AFV* (*r* = 0.8128, 95% CI: [0.7095 to 0.8819], *p* < 0.0001). Additionally, other genes demonstrated highly significant positive correlations, including *DDIAS* (*r* = 0.7255, 95% CI: [0.5852 to 0.8237], *p* < 0.0001), *SKP2* (*r* = 0.7193, 95% CI: [0.5766 to 0.8194], *p* < 0.0001), *SMARCA4* (*r* = 0.6998, 95% CI: [0.5498 to 0.8061], *p* < 0.0001), *FOXM1* (*r* = 0.6735, 95% CI: [0.5140 to 0.7880], *p* < 0.0001),

*AGO2* (*r* = 0.6647, 95% CI: [0.5022 to 0.7818], *p* < 0.0001) and *UCHL5* (*r* = 0.6511, 95% CI: [0.4841 to 0.7724], *p* < 0.0001).

Negative Associations (Blue saturation): In contrast, a specific set of transcripts displayed a significantly strong inverse linear correlation with *MKI67*, notably *ESR1* (*r* = -0.4796, 95% CI: [-0.6477 to -0.2669], *p* < 0.0001), *PGR* (*r* = -0.4072, 95% CI: [-0.5923 to -0.1813], *p* = 0.0008), *CXXC5* (*r* = -0.3657, 95% CI:-0.5597 to -0.1337], *p* = 0.0027) and *RARA* (*r* = -0.3327, 95% CI: [-0.5333 to -0.09665], *p* = 0.0068). This group also includes *ERBB4* (*r* = -0.2792, 95% CI: [-0.4897 to -0.03785], *p* = 0.0243), and *FOXA1* (*r* = -0.2802, 95% CI: [-0.4906 to -0.03895], *p* = 0.0238). Non-Significant Interactions (White cells): Finally, *PYGO1* (*r* = 0.1807, 95% CI: [-0.06613 to 0.4067], *p* = 0.1498), *H2AFZ* (*r* = 0.07614, 95% CI: [-0.1709 to 0.3142], *p* = 0.5466), *PPARA* (*r* = 0.04107, 95% CI: [-0.2049 to 0.2821], *p* = 0.7453), as well as *SHC2* (*r* = -0.1792, 95% CI: [-0.4054 to 0.06766], *p* = 0.1532), *GATA3* (*r* = -0.1509, 95% CI: [-0.3808 to 0.09652], *p* = 0.2301), *GFRA1* (*r* = -0.1473, 95% CI: [-0.3776 to 0.1002], *p* = 0.2418) and *SFRP1* (*r* = -0.07137, 95% CI: [-0.3099 to 0.1756], *p* = 0.5721) exhibited transcriptional independence regarding *MKI67* levels, presenting coefficients near zero and *p*-values that exceeded the statistical screening threshold (*p* = 0.05).

Consistent with findings from the previous matrix, an additional correlation analysis examining relationships at the protein level between IHC-derived Ki-67 values and the 34 genes from the nuclear module (**Supplementary Table 6**) was performed. The strongest significant positive associations were shown by genes associated with proliferation and chromatin: *CDK6* (*r* = 0.3962, 95% CI: [0.1559 to 0.5922], *p* = 0.0019), *SKP2* (*r* = 0.3699, 95% CI: [0.1257 to 0.5718], *p* = 0.0039), *DDIAS* (*r* = 0.3367, 95% CI: [0.08819 to 0.5457], *p* = 0.0091) and *CCNE1* (*r* = 0.3287, 95% CI: [0.07929 to 0.5394], *p* = 0.011). *CCNE1, DDIAS* and *SKP2* were consistently identified as genes positively and significantly correlated with Ki-67/*MKI67* in both analyses, at the mRNA and protein levels.

On the other hand, negative correlations were identified in hormonal signaling-associated genes: *ESR1* (*r* = -0.4915, 95% CI: [-0.6640 to -0.2694], *p* < 0.0001), *CXXC5* (*r* = -0.4382, 95% CI: [-0.6242 to -0.2051], *p* = 0.0005), *FOXA1* (*r* = -0.4231, 95% CI: [-0.6128 to -0.1873], *p* = 0.0008) and *PGR* (*r* = -0.3793, 95% CI: [-0.5792 to -0.1365], *p* = 0.003). *ESR1, CXXC5, PGR* and *FOXA1* were consistently identified as genes negatively and significantly correlated with Ki-67/*MKI67* in both analyses, at the mRNA and protein levels.

### 3.10 Comparative analysis of the 34-gene module and BC genomic signatures

To provide biological context for the identified 34-gene module, a comparative analysis with established BC genomic signatures was performed. The comparison highlighted both shared and distinct biological characteristics among the evaluated signatures (**Supplementary Table 7**, **Supplementary Material 2**).

### 3.11 Proposed signaling mechanisms of the ERα-E2F/FOXM1 axis

**Figure 5:**
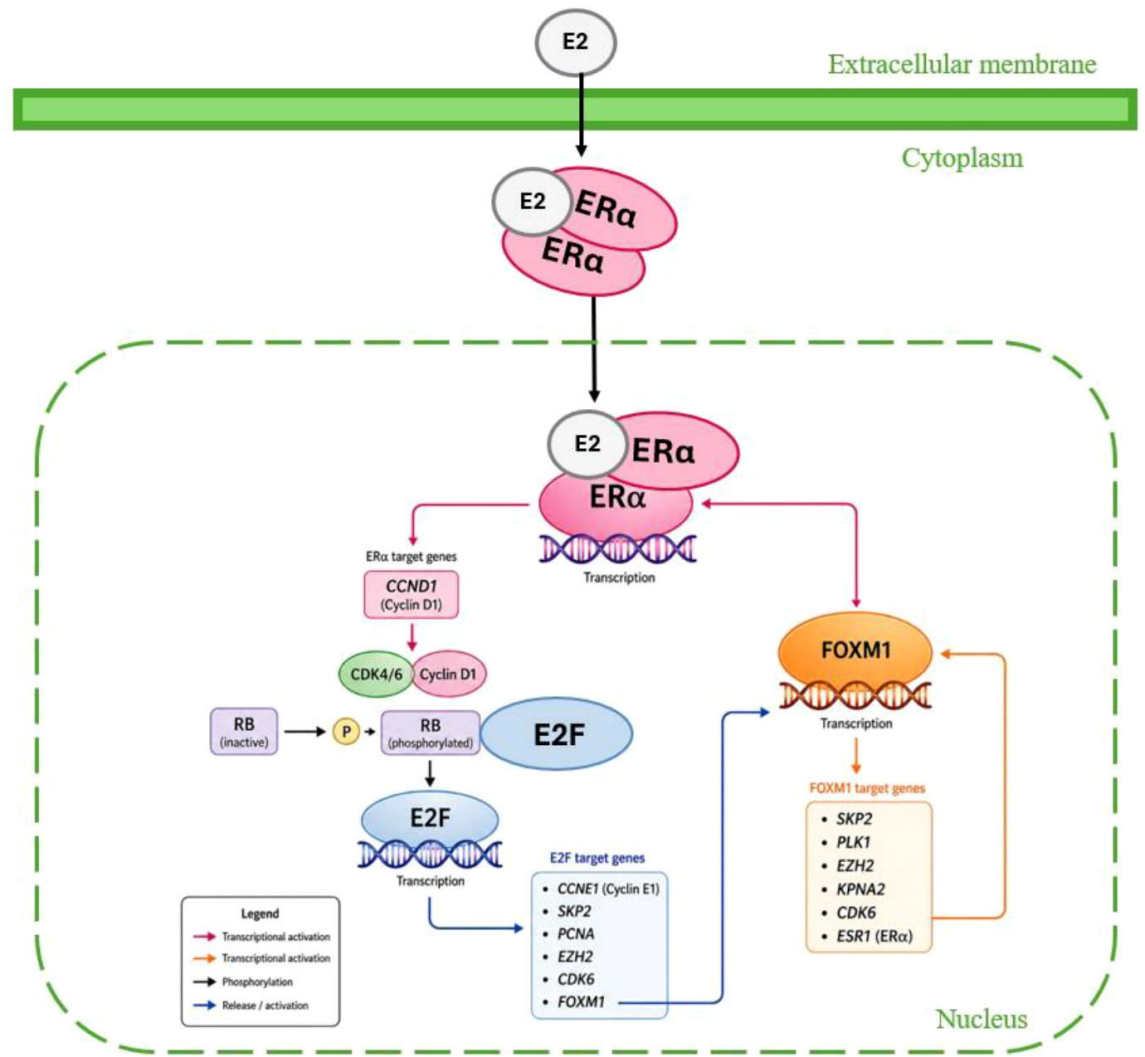
Proposed signaling of the ERα-E2F/FOXM1 potential regulatory axis driving the identified 34-gene signature in BC. The figure shows the suggested interrelated signaling pathways of ERα (hormonal signaling), E2F/FOXM1 (proliferation), and together, transcriptional regulation. Mechanisms are based on data from Paternot *et al*., 2010, Millour *et al.*, 2010 & 2011 & Sanders *et al.*, 2013. Figure generated with the assistance of ChatGPT (OpenAI) and subsequently refined by the authors. E2: Estrogen/estradiol; CDK4/6: Cyclin-Dependent Kinases 4 and 6; RB: retinoblastoma protein.

**Figure 5** summarizes the proposed interaction behind the ERα-E2F/FOXM1 potential regulation. ERα has been reported as an indirect effector of E2F activity (via cyclin D), which in turn regulates the transcription of *FOXM1*. Consequently, FOXM1, as a master regulator transcriptional factor, allows for transcription of proliferative genes and cell cycle progression in BC. Curiously, a feed-forward loop emerges from the interaction between FOXM1 and ESR1, as they can regulate each other (10, 11, 36, 37). Notably, these three genes and their pathways are interrelated, which might be beneficial for therapeutic targeting.

## 4 Discussion

BC classification depends largely on ERα expression. As a complex network of malignant crosstalk events, the ERα cascade has been of great interest and relevance in BC. However, the complete list of participants has not yet been fully unraveled (38). Therefore, the current study aimed to investigate the ERα signaling pathway in depth and identify nuclear markers that reflect the true status of ERα transcriptional activity across different BC groups. A 34-nuclear gene regulatory module was identified by contrasting the expression of PAM50-based subtypes. ERα-E2F/FOXM1 was identified as a potential axis driving proliferation, and, alongside hormonal signaling and transcriptional regulation, their expression patterns would contribute to precise BC stratification.

This project performed a PAM50 gene test on the expression profiles, enabling more uniform and accurate classification (5, 14). Of the 65 samples, 25 (38%) were assigned to a new subtype. Basal tumors had the highest number of samples reclassified into another subtype, mainly to LumA. Both HER2+ and LumB samples were also reassigned as LumA. Consistently, LumA cases were the least reclassified, and most of these samples were then listed as LumB (**Supplementary Table 1**). Some studies (6, 39) have shown a similar pattern of misclassification between Lum A and B samples, probably due to variations in the Ki-67 proliferation index (LumA <20% and LumB ≥20%) and HER2 status (LumA is HER2+ and LumB is HER2+/-). Moreover, some reports have shown that ER-low cases (≤1-10% of ER expression in tumor cells by IHC) can be easily misclassified as ER-negative (HER2+ or mostly TN), which could explain the common misclassification of TN to ER-positive or vice versa (40, 41).

Several studies have reported significant discordance between IHC-based surrogate classification and molecular intrinsic subtypes by PAM50 with rates ranging from roughly 30% to over 40%, depending on the cohort and subtype analyzed (6). Consistently, in our results, out of a total of 65 samples classified by IHC, 25 samples (corresponding to 38%), were stratified by PAM50 into a new subtype. It is worth mentioning that the discrepancy between IHC and PAM50 subtyping mainly lies in the fact that IHC only provides a simplified surrogate estimate of BC subtypes, by evaluating expression of individual protein markers (ER, PR, HER2, and KI-67); while PAM50 analyzes broader transcriptomic programs that reflect the tumor molecular landscape.

Additionally, we found in **Table 1** that PAM50-based subtypes differed by menopausal status (C*hi*-squared=11.27, *p*-value=0.0237). Commonly, LumA tumors tend to prevail in postmenopausal women, whereas luminal B, HER2-enriched, and basal-like tumors (aggressive phenotype) are relatively more frequent in premenopausal patients (42). However, in our cohort, LumB and HER2-enriched tumors were more frequent among postmenopausal patients. This discrepancy may be explained by the limited sample size and the unequal distribution of molecular subtypes within our dataset. These findings should be interpreted with caution, as the limited sample sizes in the PAM50 subgroups (ranging from 4 to 25 samples) may have reduced statistical power and hindered the detection of additional significant associations. Therefore, observed subtype-specific patterns require confirmation in larger independent cohorts.

PAM50 is a widely used molecular classification tool that provides biological stratification to breast tumors; nonetheless, its interpretation should consider the complexity of tumor biology and intratumoral heterogeneity (43). Despite the limited representation of some subgroups in our cohort, PAM50 classification allowed the identification of subtype-associated expression patterns in the heatmaps. These patterns were broadly consistent with the functional annotation of the identified gene groups, although individual genes showed variability across molecular subtypes.

While a plethora of studies have investigated the ERα signaling pathway (44–48) our research took a broader approach, retrieving not only genes already associated with the ERα pathway in curated signaling databases and/or the literature, but also potentially associated genes found in journal publications that have not yet been added to the databases as well (**Figures 1A, B**). The 2012–2022 timeframe was selected to focus on the most recent and methodologically comparable evidence regarding genes associated with, or potentially associated with, ERα. During this period, substantial advances in genomic and transcriptomic technologies, including next-generation sequencing and high throughput expression profiling, have improved the identification and characterization of ERα-related genes. It is worth noting that relevant genes may have been reported before 2012; however, many of these genes were subsequently re-evaluated, validated, or revisited in publications during the selected period.

The comparison between the literature and the pathway-oriented platforms revealed Erα-associated genes already implicated in BC. Estrogen receptors (*ESR1* and *ESR2*), ERα cofactors and transcriptional regulators (SP1, FOS, JUN & HSP90AA1), ERα target genes (*BCL2*, *CCND1* and *MMP2*) as well as genes from ER-associated oncogenic pathways (PIK3CA, AKT1, IGF1R & SHC1) were found (4). Furthermore, genes showing evidence of functional or regulatory associations with ERα signaling were retrieved from the literature, such as *FOXM1*, *SKP2*, *PLK1*, *SMARCA4* and *EZH2*. Interestingly, *FOXM1, SKP2, EZH2* and *PLK1* are targets of the E2F transcription factor family and are associated with cell cycle progression, whereas *SMARCA4* has been suggested as an E2F regulator. By prioritizing experimentally validated interactions, this study provides evidence for the reliability of the results presented.

The supervised analysis was restricted to prioritize upregulated genes encoding proteins with reported nuclear subcellular localization. Considering that ERα functions primarily as a nuclear transcription factor, proteins related with nuclear processes may represent potential regulators of ERα transcriptional activity. From the supervised analysis of the 490 nuclear genes in our database and the 65 expression profiles of Mexican BC patients (**Figures 2A, B**), we identified 34 genes with FDR-adjusted *p*-values <0.0001 (**Supplementary Table 3**). Applying a stringent FDR threshold allowed us to reduce false positives in identifying the most statistically significant genes (49).

Gene set was divided into groups according to function (1, 2, and 3) and expression (A, B, C, and D). Functional activity (1. Hormonal signaling, 2. Proliferative/cell cycle regulation, and 3. Genome regulation and cancer-related processes) as well as expression pattern across BC subtypes (A. general upregulation, B. upregulation in proliferative subtypes and downregulation in non-proliferative tumors, C. upregulation in hormonal and normal subtypes and downregulation in proliferative subtypes, as well as D. general downregulation) were contrasted. Interestingly, differential expression across molecular subtypes was associated with functional biological background. Genes (*PARP1, SMARCA4, PCNA, CHAF1A, H2AFX, H2AZ1, H2AZ2 and UCHL5*) included in the genome regulation and cancer-related processes module 3 showed notorious upregulation in all the subtypes (module A). In the same way, genes (*GFRA1, ESR1, GATA3, ERBB4, FOXA1, SHC2* and *PGR*) linked with hormonal signaling (module 1) were enriched for LumA, LumB and normal-like (in different degree), but not for HER2-enriched and basal samples (module C). Conversely, genes (*CCNE1, E2F2, FOXM1, PLK1* and *SKP2*) that are associated with proliferation and/or cell cycle regulation (module 2) in BC, show an upregulated pattern displayed by LumB, HER2-enriched, and basal subtypes, but not LumA and normal-like (module B) subtypes, reported as aggressive and proliferative.

Further analyses explored biological processes and transcription factor enrichment (**Figures 3A, B**) in our panel. The most enriched biological activities (**Supplementary Table 4**) were summarized in transcription, chromatin, and cell cycle regulation in addition to hormonal signaling. Moreover, transcription factor enrichment analyses using ChEA dataset revealed a significant enrichment of targets of the E2F family and FOXM1; approximately 30% of the genes within our set correspond to members (E2F2 and E2F3), known targets or regulators (*FOXM1, CCNE1, SKP2, PLK1, PCNA, EZH2, KPNA2* and *CDK6*) of the *E2F* transcription factor family (37, 50, 51). Regarding ERα enriched target genes, our set includes established targets (*PGR, GATA3, FOXA1, ERBB4, SFRP1, RARA,* and *CXXC5*), proliferation-associated genes likely regulated indirectly by collaboration with E2F/FOXM1 (*CCNE1, CDK6* and *PLK1*), as well as downstream effectors involved in chromatin remodeling and DNA replication (*EZH2, PCNA* and *CHAF1A*) (4, 46). Hence, the functional enrichment analyses together with the supervised expression analysis supports the hypothesis that the 34 DEGs might form a nuclear module involved in estrogen-dependent transcriptional regulation associated with E2F and FOXM1. By associating function with expression across BC subtypes, the DEGs became a coordinated set of genes with biological coherence.

Thus, this nuclear module could potentially be led by the ERα-E2F/FOXM1 axis (possibly via *ESR1, PGR, FOXA1, GATA3, E2F2, E2F3, FOXM1*, and *CCNE1*), by integrating hormonal signaling (ERα), transcriptional and cell cycle regulation (E2F), as well as cell proliferation (FOXM1). Based on the previous, we suggest that this 34-gene panel could be potentially useful for obtaining a more accurate indication of differential ERα transcriptional activity across BC molecular subtypes.

Moreover, protein–protein interaction network analysis using STRING (**Figure 3C**) revealed that the identified genes form a strong and significantly (PPI enrichment p<1.0×10⁻¹^6^ and interacting score ≥0.4) interconnected functional module (**Supplementary Table 5**) rather than a random gene set (52). Mares Quiñones and colleagues (53) used STRING to identify five gene modules with similar co-expression patterns and hub genes significantly associated with each BC subtype. Their study states that PPI analysis enables the identification of functional protein interaction networks involving proteins encoded by relevant genes and the elucidation of biological mechanisms useful for the discovery of potential biomarkers or therapeutic targets in BC.

Previous studies have independently demonstrated that ESR1 modulates FOXM1 expression and vice versa (10, 11) and that E2F transcription factors control FOXM1 as part of the cell cycle transcriptional program (12, 37). However, the integration of this regulatory circuitry within molecular BC subtype stratification has not been explored before. Our results suggest that the ERα-E2F/FOXM1 transcriptional axis may contribute to the transcriptional differences underlying molecular BC subtype differentiation (**Figure 3D**). Hormonal signaling seems to be interrelated with proliferation, creating a potential expected pattern for all the subtypes; LumA: High hormonal signaling and low proliferation; LumB: high hormonal signaling and high proliferation; HER2-enriched: low-moderate hormonal signaling and high proliferation; Basal: low hormonal signaling and very high proliferation; normal-like: moderate hormonal signaling and low-moderate proliferation. A great benefit could be potentially obtained by all BC patients, especially those currently classified by IHC as ER-low or HER2-low (40, 41), which commonly have a higher probability of misclassification and treatment inefficacy.

To further support the biological relevance of the identified transcriptional module, the expression of three representative genes from the 34-gene set (**Figure 4A**) was experimentally evaluated (**Figure 4B**) in BC cell lines (MCF7, HCC1428, HCC1954, and MDA-MB-231). *E2F2* was found to be highly abundant in HCC1954, a HER2-enriched cell line, consistent with our placement of E2F2 in groups 2 and B. Moreover, the study of Lee *et al*., describes the abundance of *E2F2* in HER2+ cell lines (including HCC1954) and their proliferative activity (54). On the other hand, PARP1 was shown as highly expressed in the luminal An MCF7 cell line. Consistently, *PARP1* has been described as enabling chromatin remodeling and enhances transcription of ERα target genes in an estrogen-dependent manner (55, 56), which would explain its high expression patterns in all the BC subtypes, especially in LumA (groups A and 3).

In contrast, *PAK2* expression showed heterogeneous patterns across PAM50 subtypes and did not fully align with the global expression pattern associated with the assigned group. *PAK2* was included in groups corresponding to genome regulation and cancer-related processes, as well as proliferative-associated expression patterns (3 and B) identified in the BC patient expression profiles; however, individual *PAK2* expression showed variability among samples, including a subset of LumA tumors with higher expression levels. Consistently, the highest expression of *PAK2* in the RT-qPCR was detected in MCF7 cells. These findings suggest that *PAK2* expression may vary according to the molecular context of breast tumors and experimental models. Even though BC cell lines are useful for studying molecular subtypes, they may undergo transcriptional and phenotypic adaptations during long-term culture and do not fully capture the complexity and heterogeneity of primary tumors, including interactions with the tumor microenvironment (57, 58). Notably, *PARP1* and *PAK2* showed higher expression in the MCF7/LumA subtype in the cell line model. Both genes have been associated with endocrine therapy resistance in ER+ BC, as well as other genes in the module, such as *DDIAS* and *UCHL5*; while the former has been previously found overexpressed in tamoxifen-resistant BC cell lines; inhibitors of the latter and PSMD14 (b-AP15 and PtPT) show therapeutic potential for resistant cases (34, 59–61). *PAK2, PARP1, DDIAS* and *UCHL5* have been included in the same functional group 3; hence, potential common resistance-associated mechanisms warrant further investigation.

According to expression patterns observed in the transcriptomic dataset, *E2F2, PARP1*, and *PAK2* have been associated with proliferation, ERα signaling, and transcriptional regulation, which are relevant components of the proposed ERα-E2F/FOXM1 axis. Overall, these findings provide supportive experimental evidence partially consistent with the transcriptional patterns observed in the gene expression dataset.

In addition, some methodological considerations regarding the RT-qPCR analysis should be mentioned. Although GAPDH has been widely used as a reference gene in RT-qPCR experiments of BC studies (62, 63), we acknowledge that the stability of *GAPDH* was not formally validated across the four BC cell lines included in this study. Nonetheless, raw *GAPDH* Ct values as well as the corresponding graph for all cell lines are included in **Supplementary Figure 1**. Furthermore, since our objective was to compare normalized expression patterns across BC cell lines rather than quantify fold changes relative to a defined reference condition, the ΔCt approach was selected (64). Additionally, our study design did not include a single cell line that could serve as a biological control or calibrator suitable for ΔΔCt analysis. Accordingly, the RT-qPCR results provide supportive evidence consistent with transcriptomic observations rather than definitive quantitative validation of differential expression across BC cell lines. Future studies, including validation of housekeeping genes and implementation of additional normalization strategies, will be primordial to further validate the present findings.

The present study provides an initial characterization of the 34-gene module; however, comprehensive validation of the complete signature requires additional independent cohorts and quantitative evaluation in primary tumor datasets. The use of BC cell lines enabled experimental assessment of selected genes but should be interpreted as complementary evidence, given the known limitations of cell line models in capturing the full heterogeneity of primary tumors. In this context, complementary analyses to explore the biological consistency of the module across independent datasets may provide additional support for its underlying transcriptional structure.

Accordingly, an expression reproducibility analysis was conducted in an independent BC cohort, GSE18229 (**Supplementary Figure 4**). *SFRP1, PGR,* and *FOXA1* showed discriminatory expression patterns across the PAM50-based subtypes, which is consistent with their involvement in hormone receptor signaling and BC subtype biology (65–67). Together, these genes illustrate distinct but interconnected components of the transcriptional program captured by the 34-gene nuclear module, encompassing estrogen receptor chromatin accessibility, downstream hormone signaling activity, and modulation of proliferative and differentiation-associated pathways. Overall, these findings support the biological coherence and potential relevance of the 34-gene nuclear module in BC transcriptional regulation. Although expression patterns of certain module components were reproducible in an independent BC cohort, additional validation approaches are warranted to further establish the robustness of the complete signature.

To address this point, an expression correlation matrix between the 34-gene module and *MKI67/*Ki-67 was computed in R (**Supplementary Table 6**). The directed co-expression profile against *MKI67* clearly demonstrates the classic molecular divergence between active cell proliferation programs and the preservation of luminal differentiation in BC (**Supplementary Figure 5**). These findings reveal a distinct transcriptional antagonism: on one hand, the synchronous activation of cell cycle regulators and epigenetic modifiers, and on the other, the coordinated repression of the hormone receptor axis.

The robust positive correlation observed between *MKI67* and *EZH2* (*r* = 0.8276, p < 0.0001) represents the strongest association within our matrix, aligning with the established biological role of *EZH2* as the primary catalytic component of the Polycomb Repressive Complex 2 (PRC2). In breast oncology, *EZH2* overexpression runs parallel to high mitotic rates because it mediates histone H3 lysine 27 trimethylation (H3K27me3), a repressive epigenetic mark that target-silences critical tumor suppressors and cell cycle checkpoints, including the *CDKN2A* locus (p16/p14).

Our statistical analysis reveals a significant correlation between *EZH2* transcription and tumors characterized by a high proliferative velocity. This finding aligns with current clinical literature, where elevated levels of this transcript are frequently associated with aggressive, dedifferentiated, and poor prognosis phenotypes, particularly within high-grade LumB and TN subtypes. These data suggest that *EZH2* overexpression may reflect or contribute to the highly proliferative nature of these malignancies, warranting further functional validation to elucidate its potential role as a biomarker or therapeutic target.

In direct contrast to the proliferation block, our results demonstrate a significant inverse correlation of *MKI67* with *ESR1* (*r* = -0.4796, *p* < 0.0001) and *PGR* (*r* = -0.4072, p = 0.0008). This negative correlation signature (represented by the blue saturation in the heatmap **Supplementary Figure 5**) illustrates the phenomenon of transcriptional dedifferentiation: as the tumor acquires accelerated proliferative kinetics, the expression of mature luminal lineage markers is progressively attenuated. The loss or reduction of *ESR1* and its downstream target gene *PGR* is not merely a passive byproduct of an increased mitotic rate. A direct molecular crosstalk mechanism is well-documented in preclinical models: the hyperactivation of *EZH2* actively induces epigenetic silencing of the *ESR1* promoter through direct *PRC2* recruitment and subsequent chromatin condensation. When *ESR1* is repressed, the estrogen-dependent signaling required for normal mammary epithelial differentiation collapses. This drives a phenotypic transition toward a more primitive, cancer stem cell-like state, leading to intrinsic resistance to conventional endocrine therapies.

The identification of this divergent signature (*EZH2* “high”/*ESR1*–*PGR* “low”) within our dataset underscores the potential value of the EZH2 transcriptional load as an aggressive biomarker that complements or surpasses traditional histological Ki-67 scoring. Furthermore, from a therapeutic perspective, these results provide a strong biological rationale for exploring pharmacological EZH2 inhibitors (such as tazemetostat or GSK126) as a viable strategy for tumor resensitization. Inhibiting the methyltransferase activity of EZH2 in highly proliferative tumors could reverse the epigenetic blockade on *ESR1*, forcing the re-expression of hormone receptors and opening a therapeutic window for combinatorial endocrine regimens (68–71).

To further contextualize the biological characteristics of our 34-gene module, we compared it with established BC genomic signatures (**Supplementary Table 7**), including EndoPredict, Oncotype DX, Prosigna (*PAM50*), and MammaPrint. Even though our module shares only a limited number of genes with established genomic assays, all signatures converge on common biological processes, particularly estrogen receptor signaling, cell cycle regulation, and proliferation. This limited gene overlap may suggest that distinct gene sets can capture similar biological programs.

Unlike commercially available signatures, our 34-gene module was derived from genes associated with ERα signaling, rather than from prognostic gene selection. Although exploratory, the enrichment analyses and expression patterns suggest that coordinated biological processes related to hormone signaling, cell-cycle modulation, and transcriptional regulation, as well as the E2F/FOXM1 regulatory network, may contribute to the molecular distinction among PAM50 BC subtypes. Our results suggest that the coordinated expression of biologically related genes may better reflect the transcriptomic programs underlying molecular subtype differentiation. These observations provide a biologically plausible framework for investigating subtype discrimination but require functional validation to elucidate the associated regulatory mechanisms.

Taken together, our results provide evidence of a 34-gene nuclear program forming a highly interconnected network enriched for FOXM1/E2F signaling components. This nuclear module integrates hormone signaling, cell cycle progression, proliferation, and genome regulatory processes, all closely linked to ERα activity (**Figure 5**). In this context, analyzing the interrelationships among these key oncogenic pathways may better reflect the functional status of ERα signaling across BC subtypes (72, 73).

Thus, we propose the 34-gene panel as a candidate transcriptomic framework that may provide insights into BC subtype-associated biology by capturing tumor contrasts related primarily to proliferation and ERα signaling. Since most previous studies have addressed the axis separately, our study serves as a starting point for considering the ERα-E2F/FOXM1 network as a potential biological framework for future investigations of subtype characterization and therapeutic hypotheses. Nevertheless, larger cohorts and additional experimental validation are required to determine whether this transcriptional module may have potential utility as a biomarker signature for BC stratification.

## Data Availability

The data analyzed in this study is subject to the following licenses/restrictions: The raw data supporting the conclusions of this article will be made available by the authors, without undue reservation. Requests to access these datasets should be directed to rortizl@tec.mx.
