# Supplementary material for: Identification of a nuclear transcriptional module associated with ERα and E2F/FOXM1 signaling across PAM50 breast cancer subtypes
Source: Front Oncol. 2026 Aug 5;16:1874614. doi: 10.3389/fonc.2026.1874614 (PMC13485578; doi:10.3389/fonc.2026.1874614)
Supplement: Supplementary file 1 [file DataSheet1.docx]

Supplementary Material

# 1 Supplementary Figures and Tables

**Ct**

**Figure S1.** *GAPDH* expression across the four BC cell lines. *GAPDH* transcript levels were evaluated across four breast cancer cell lines representing different PAM50-classified subtypes. The X axis represents each BC cell line, and the Y axis corresponds to Ct values obtained by RT-qPCR. LA: luminal A; LB: luminal B; HER2: HER2+ and TN: Triple negative.

**(A)**

**(B)**

**Figure S2 (A-B).** Principal component analysis (PCA) of the 65 BC samples for batch effect investigation. Exploratory visualization of sample clustering trends for the BC samples collected in two time periods: 2011-2014 (blue figures) and 2017-2019 (red figures). The percentage of total variance and per component (PC1, PC2 and PC3) is shown in bold. (**A**) PCA with no shape by condition. (**B**) PCA shaped by condition (PAM50-based subtypes). Outliers are shown separately from the main groups. CHP refers to an Affymetrix data file format. Plots obtained with TAC software (Affymetrix). LA: luminal A; LB: luminal B; HER2: HER2+; Basal: Basal-like; Normal: Normal-like.

**(A)**

**(B)**

**Figure S3 (A-B)** Heatmaps from the supervised analysis showing expression patterns of the complete gene set. (**A)** Heatmap of the first half of the 490 genes (n=245), prior to statistical filtering. (**B)** Heatmap of the second half of the 490 genes (n=245), prior to statistical filtering. Expression by subtype (condition). Blue cells represent low gene expression and red corresponds to high gene expression. Each column is a patient, and each row is a gene (blank space is due to a high quantity of genes and a limited space). Heatmaps obtained with TAC software (Affymetrix). LA: luminal A; LB: luminal B; HER2: HER2+; Basal: Basal-like; Normal: Normal-like.

**Figure S4.** Hierarchical clustering analysis showing the expression reproducibility of the 34- gene nuclear module in an independent breast cancer cohort (GSE18229). Heatmap illustrating gene expression data from 199 PAM50-classified samples analyzed using the SurvExpress platform and database. Color key is shown in the left upper corner of the figure: red indicates overexpression and green represents downregulation (p >0.05). Columns represent individual patients, and rows represent genes. The Id number is shown next to the Gene symbol. Gene symbols in the heatmap correspond to the annotations provided by the SurvExpress platform.

**Figure S5.** Expression correlation matrix of the 34-gene module and *MKI67*. Heatmap displaying co-expression relationships among the analyzed genes. Values within each cell represent the observed Pearson correlation coefficient (*r*). The color code simultaneously reflects the direction of the association and its statistical significance (p < 0.05): red indicates a significant positive correlation, blue indicates a significant negative correlation, and color intensity is proportional to the statistical strength of the finding. White cells represent non-significant correlations (p >0.05).

| Table S1. Original BC subtyping by IHC and reclassification with PAM50 | | | | | | | | | |
| --- | --- | --- | --- | --- | --- | --- | --- | --- | --- |
| Serial number | **Sample ID** | **IHC subtype** | **PAM50 subtype** | **Reclassified** | **Serial number** | **Sample ID** | **IHC subtype** | **PAM50 subtype** | **Reclassified** |
| 1 | S_03 | TN | Basal | No | **34** | S_59 | TN | LumA | Yes |
| 2 | S_06 | TN | Basal | No | **35** | S_64 | LumA | LumA | No |
| 3 | S_07 | Her2 | LumA | Yes | **36** | S_66 | LumA | LumA | No |
| 4 | S_08 | TN | Basal | No | **37** | S_69 | TN | Basal | No |
| 5 | S_11 | LumB | LumB | No | **38** | S_70 | TN | Basal | No |
| 6 | S_14 | Her2 | LumA | Yes | **39** | S_73 | Her2 | LumA | Yes |
| 7 | S_15 | Her2 | Her2 | No | **40** | S_80 | LumA | LumB | Yes |
| 8 | S_16 | LumB | LumB | No | **41** | S_96 | TN | Basal | No |
| 9 | S_19 | LumA | Normal-like | Yes | **42** | S_113 | TN | Basal | No |
| 10 | S_20 | LumA | LumA | No | **43** | S_121 | TN | Basal | No |
| 11 | S_21 | LumB | LumA | Yes | **44** | S_125 | Her2 | Normal-like | Yes |
| 12 | S_24 | NA | LumB | Yes | **45** | S_128 | TN | LumA | Yes |
| 13 | S_25 | LumA | LumA | No | **46** | S_134 | TN | Basal | No |
| 14 | S_26 | TN | Basal | No | **47** | S_137 | TN | LumA | Yes |
| 15 | S_27 | LumB | LumA | Yes | **48** | S_141 | TN | Basal | No |
| 16 | S_28 | LumA | LumA | No | **49** | S_152 | TN | LumA | Yes |
| 17 | S_29 | LumA | LumA | No | **50** | S_160 | TN | LumB | Yes |
| 18 | S_31 | LumA | LumA | No | **51** | S_161 | TN | Normal-like | Yes |
| 19 | S_32 | Her2 | Her2 | No | **52** | S_174 | TN | Basal | No |
| 20 | S_33 | TN | Basal | No | **53** | S_180 | TN | Basal | No |
| 21 | S_34 | TN | Basal | No | **54** | S_225 | TN | Her2 | Yes |
| 22 | S_36 | LumA | LumB | Yes | **55** | S_016* | TN | Her2 | Yes |
| 23 | S_37 | LumA | LumA | No | **56** | S_026* | TN | Her2 | Yes |
| 24 | S_38 | TN | LumA | Yes | **57** | S_031* | TN | Basal | No |
| 25 | S_39 | LumB | LumA | Yes | **58** | S_032* | TN | Basal | No |
| 26 | S_40 | Her2 | LumA | Yes | **59** | S_042* | TN | LumA | Yes |
| 27 | S_41 | LumB | LumB | No | **60** | S_055* | TN | Basal | No |
| 28 | S_44 | LumB | LumB | No | **61** | S_056* | TN | Basal | No |
| 29 | S_47 | TN | Basal | No | **62** | S_119* | TN | Basal | No |
| 30 | S_48 | TN | Normal-like | Yes | **63** | S_136* | TN | Basal | No |
| 31 | S_52 | TN | Basal | No | **64** | S_138* | TN | Basal | No |
| 32 | S_53 | TN | LumA | Yes | **65** | S_140* | TN | Basal | No |
| 33 | S_58 | LumA | LumA | No |  |  |  |  |  |
| *Samples corresponding to sample collection carried out during 2017-2019 (n=11). Remaining samples were collected during 2011-2014 (n=54). | | | | | | | | | |

| Table S2. PAM50-based subtype distribution across the two sample collection periods (2011-2014 and 2017-2019) | | | |
| --- | --- | --- | --- |
| Subtype | **Sample distribution by collection period** | | **Total samples by subtype** |
|  | **2011-2014** | **2017-2019** |  |
| LumA | 22 | 1 | = 23 |
| LumB | 8 | 0 | = 8 |
| HER2-enriched | 3 | 2 | = 5 |
| Basal-like | 17 | 8 | = 25 |
| Normal-like | 4 | 0 | = 4 |
|  |  | **Total samples** | 65 |

| Table S3. Significant DEGs (fold change ≥ 2 or ≤ −2, FDR<0.0001) from the supervised analysis amongst BC subtypes | | | | | | |
| --- | --- | --- | --- | --- | --- | --- |
| Number | **Gene symbol** | **Gene name** | **Adjusted p-value FDR <0.001** | **Potential or established association with the ERα** | **Reference** |  |
| Group 1. Hormonal signaling | | | | | |  |
| 1 | ESR1 | Estrogen receptor 1 | 1.49E-07 | - A primordial hormonal marker in BC. Participates in BC development and endocrine treatment resistance. | (3) |  |
| 2 | PGR | Progesterone receptor | 2.81E-05 | - PGR is an ERα transcriptional target. Its transcription is estrogen dependent. | (66) |  |
| 3 | FOXA1 | Forkhead Box A1 | 4.91E-08 | - FOXA1 has been indicated as a coactivator in ERα transcriptional activity and promoter of endocrine resistance in BC. | (67) |  |
| 4 | GATA3 | GATA Binding Protein 3 | 9.75E-05 | - GATA3 is a pioneer factor that works together with ERα and FOXA1 in breast normal and malignant development. | (74) |  |
| 5 | ERBB4 | Erb-b2 receptor tyrosine kinase 4 | 1.67E-05 | - Enhances ERα transcriptional activity in BC cells. ESR1/DNAJC12/ERBB4 is the suggested modulatory network. | (75) |  |
| 6 | GFRA1 | GDNF family receptor alpha 1 | 4.36E-05 | - GFRA1 was described as an ERα-regulated gene in BC. - Along with RET, they activate MAPK and AKT signaling in ER+ BC cells. | (76,77) |  |
| 7 | SFRP1 | Secreted Frizzled Related Protein 1 | 6.28E-05 | - Antagonist of WNT signaling. When SFRP1 is silenced, the ERα signaling increases, possibly via a Wnt/β-catenin-dependent mechanism. | (65) |  |
| 8 | RARA | Retinoic Acid Receptor Alpha | 4.78E-05 | - RARA has been described with contrasting roles in BC. RARA, along with retinoid X receptor, can disrupt transcription via ERα. Also, RARA has a role in ERα cofactor binding. | (78) |  |
| 9 | PPARA | Peroxisome proliferator-activated receptor alpha | 3.08E-05 | - PPARA and ERα are inversely expressed in BC. ERα can modulate PPARA levels. | (79) |  |
| 10 | CXXC5 | CXXC finger protein 5 | 7.91E-07 | - CXXC5 is regulated by ERα in BC. | (80) |  |
| 11 | SHC2 | SHC adaptor protein 2 | 3.42E-05 | - Associated with breast oncogenesis and endocrine resistance (through EGFR signaling) in BC pathways in signaling databases. | (81) |  |
| Group 2. Proliferative/cell cycle | | | | | |  |
| 12 | CCNE1 | Cyclin E1 | 7.81E-08 | - CCNE1 is an E2F1-responsive gene, which in turn is an ERα target gene. - CCNE1 was suggested as a marker of resistance (along PLK1) to palbociclib (CDK4/6 inhibitor) and endocrine therapy in metastatic BC. | (82) |  |
| 13 | CDK6 | Cyclin Dependent Kinase 6 | 3.92E-06 | - CDK6 and CDK4 are assembled with cyclin D1 (an ERα target gene) to allow progression of cell cycle in a malignant breast environment. - Overexpressed CDK6 was proposed as a marker of resistance of CDK4/6 and ERα inhibitors in BC. | (83) |  |
| 14 | CDKN2A | Cyclin-dependent kinase inhibitor 2A | 1.33E-05 | - CDKN2A (p16 protein) opposes ERα-mediated cell cycle progression by blocking CDK4/6 function in BC. | (84) |  |
| 15 | E2F2 | E2F transcription factor 2 | 5.43E-05 | - ZMIZ1 and ERα bind to E2F2 and modulate its activity as cell cycle regulator in ER+ BC. | (51) |  |
| 16 | E2F3 | E2F transcription factor 3 | 1.41E-06 | - E2F3 was associated with endocrine, chemo, and radiotherapy resistance in BC. - The ER/CDK4/Rb/E2F axis has been described in antiestrogen-resistant breast tumor growth as well. | (37,50) |  |
| 17 | FOXM1 | Forkhead box M1 | 7.02E-12 | - FOXM1 positively regulates the activity of ERα, and it is involved in endocrine therapy resistance in BC. | (11) |  |
| 18 | PLK1 | Polo Like Kinase 1 | 4.35E-06 | - PLK1 is a regulator of ERα transcriptional activity in BC. | (32) |  |
| 19 | SKP2 | S-phase kinase associated protein 2 | 2.35E-08 | - SKP2 was shown to be an ERα regulator. Their levels appear as inversely correlated. - The ERα/SKP2/E2F-1 network has been found to cause a feed-forward loop for BC promotion via cell cycle progression. | (85,31) |  |
| 20 | EZH2 | Enhancer of zeste 2 polycomb repressive complex 2 subunit | 3.33E-11 | - EZH2-ER-GREB1 axis regulates tamoxifen resistance in BC. | (34) |  |
| 21 | KPNA2 | Karyopherin Subunit Alpha 2 | 1.18E-08 | - KPNA2 is regulated by FOXM1, which in turn is reciprocally modulated by the ERα. | (86) |  |
| Group 3. Genome regulation and cancer-related processes | | | | | |  |
| 22 | SMARCA4 | SWI/SNF related, matrix associated, actin dependent regulator of chromatin, subfamily a, member 4 | 8.32E-05 | - ERα could recruit BRG1 (also known as SMARCA4) for chromatin remodeling and increase of its transcriptional activity in BC. | (87) |  |
| 23 | CHAF1A | Chromatin assembly factor I subunit A | 3.58E-08 | - CHAF1A was mentioned as correlated with endocrine treatment resistance. | (88) |  |
| 24 | H2AFX | H2A.X Variant Histone | 7.48E-09 | - Altered H2AFX has been related to breast carcinogenesis and ERα positive status. | (89) |  |
| 25 | H2AZ1 | H2A.Z Variant Histone | 9.78E-06 | - H2AZ1 and H2AZ2 encode the H2A.Z protein, which modulates estrogen-dependent signaling by binding to the promoters of ERα target genes. | (90) |  |
| 26 | H2AZ2 | H2A.Z Variant Histone 2 | 5.98E-05 |  |  |  |
| 27 | PYGO1 | Pygopus family PHD finger 1 | 8.67E-05 | - Participates in Wnt/β-catenin signaling pathway. - WNT/β catenin axis is known to participate in endocrine resistance. | (91) |  |
| 28 | PCNA | Proliferating Cell Nuclear Antigen | 7.32E-08 | - PCNA promotes ERα binding to ERE of its target genes, increasing their basal expression in BC. | (92) |  |
| 29 | UCHL5 | Ubiquitin C-terminal Hydrolase L5 | 2.11E-06 | - The b-AP15 and PtPT are PSMD14 and UCHL5 inhibitors, can cause growth suppression of ERα-positive BC cells. | (61) |  |
| 30 | PAK2 | p21 (RAC1) activated kinase 2 | 1.24E-05 | - IGF1R promotes PAK2/PIX endocrine therapy resistance (with tamoxifen) and poor clinical outcomes in BC. - PAK2 may indirectly influence chromatin remodeling and transcriptional programs. | (93–95) |  |
| 31 | PARP1 | Poly-(ADP-ribose) polymerase 1 | 2.67E-08 | - PARP-1 could regulate the estrogen dependent ER-α and FOXA1 binding to target genes, promoting ERα+ BC. | (96) |  |
| 32 | AGO2 | Argonaute RISC Component 2 | 9.30E-07 | - AGO2 is a potential modulator of altered ERα signaling, and it enhances the expression of ERα 36-kDa variant. | (97) |  |
| 33 | DDIAS | DNA damage induced apoptosis suppressor | 4.58E-08 | - The Paraspeckle component 1 (PSPC1) has a role in post-transcriptional regulation of ESR1 and Sec1 family domain containing 2 (SCFD2). - DDIAS is a target gene of SCFD2. The PSPC1-SCFD2-DDIAS axis was found overexpressed in tamoxifen-resistant ER+ BC. | (60) |  |
| 34 | KRT16 | Keratin 16 | 1.06E-05 | - KRT16 is associated with high tumor aggressiveness, and its expression has been found as inversely proportional to the expression of ERα. | (98) |  |

| Table S4. Top 15 biological processes enriched in the GO analysis of the 34 DEGs | | | | | |
| --- | --- | --- | --- | --- | --- |
| Position | **Term** | **Adjusted**  **p-value** | **-log10(FDR)** | **Gene count** | **Genes** |
| 1 | Positive Regulation of DNA-templated Transcription (GO:0045893) | 1.86E-12 | 11.7314 | 17 | *FOXA1, H2AZ1, PARP1, CDKN2A, GATA3, FOXM1, ESR1, UCHL5, SMARCA4, SFRP1, ERBB4, AGO2, RARA, E2F2, E2F3, PGR & PPARA* |
| 2 | Positive Regulation of Transcription by RNA Pol II (GO:0045944) | 1.49E-10 | 9.8256 | 14 | *FOXA1, H2AZ1, PARP1, CDKN2A, GATA3, FOXM1, ESR1, SMARCA4, AGO2, RARA, E2F2, E2F3, PGR & PPARA* |
| 3 | Regulation of miRNA Transcription (GO:1902893) | 1.06E-09 | 8.9759 | 6 | *FOXA1, RARA, GATA3, PPARA, ESR1 & SMARCA4* |
| 4 | Positive Regulation of RNA Biosynthetic Process (GO:1902680) | 1.30E-08 | 7.8857 | 10 | *SFRP1, ERBB4, CDKN2A, RARA, GATA3, FOXM1, PPARA, ESR1, UCHL5 & SMARCA4* |
| 5 | Negative Regulation of Transcription by RNA Pol II (GO:0000122) | 1.36E-08 | 7.8671 | 11 | *PARP1, CDKN2A, PLK1, RARA, GATA3, CXXC5, FOXM1, PPARA, ESR1, SMARCA4 & EZH2* |
| 6 | Regulation of Transcription by RNA Pol II (GO:0006357) | 1.38E-08 | 7.8598 | 17 | *FOXA1, H2AZ1, PARP1, CDKN2A, PLK1, GATA3, CXXC5, FOXM1, ESR1, SMARCA4, AGO2, RARA, E2F2, E2F3, PGR, PPARA & EZH2* |
| 7 | Negative Regulation of DNA-templated Transcription (GO:0045892) | 3.27E-08 | 7.4859 | 12 | *SFRP1, PARP1, CDKN2A, PLK1, RARA, GATA3, CXXX5, FOXM1, PPARA, ESR1, SMARCA4 & EZH2* |
| 8 | Regulation of Cell Cycle (GO:0051726) | 3.68E-08 | 7.4347 | 8 | *SFRP1, CDK6, CDKN2A, PLK1, RARA, GATA3, FOXM1 & UCHL5* |
| 9 | Chromatin Organization (GO:0006325) | 4.15E-08 | 7.3824 | 8 | *FOXA1, H2AZ1, H2AZ2, CHAF1A, ESR1, UCHL5, SMARCA4 & EZH2* |
| 10 | Regulation of DNA-templated Transcription (GO:0006355) | 5.45E-08 | 7.2634 | 16 | *FOXA1, PARP1, CDKN2A, GATA3, FOXM1, ESR1, UCHL5, SMARCA4, SFRP1, ERBB4, RARA, E2F2, E2F3, PGR, PPARA & EZH2* |
| 11 | Nuclear Receptor-Mediated Signaling Pathway (GO:0141193) | 2.14E-07 | 6.6702 | 4 | *RARA, PGR, PPARA & ESR1* |
| 12 | Response to Estrogen (GO:0043627) | 2.14E-07 | 6.6702 | 4 | *SFRP1, RARA, GATA3, ESR1* |
| 13 | Response to Estradiol (GO:0032355) | 2.43E-07 | 6.6146 | 4 | *FOXA1, H2AZ1, SFRP1 & ESR1* |
| 14 | Positive Regulation of DNA Repair (GO:0045739) | 4.18E-07 | 6.3793 | 5 | *PCNA, PARP1, FOXM1, UCHL5 & SMARCA4* |
| 15 | Negative Regulation of RNA Biosynthetic Process (GO:1902679) | 7.26E-07 | 6.1391 | 8 | *SFRP1, PARP1, CDKN2A, RARA, GATA3, FOXM1, SMARCA4 & EZH2* |

| Table S5. Combined score of the interactions displayed on STRING by the 34 DEGs | | | | |
| --- | --- | --- | --- | --- |
| Interaction | **Node 1** | **Node 2** | **Combined score** | **Number of interactions** |
| 1 | AGO2 | SKP2 | 0.404 | 11 |
| 2 |  | H2AZ2 | 0.524 |  |
| 3 |  | SMARCA4 | 0.533 |  |
| 4 |  | CCNE1 | 0.548 |  |
| 5 |  | H2AZ1 | 0.556 |  |
| 6 |  | KPNA2 | 0.567 |  |
| 7 |  | H2AX | 0.584 |  |
| 8 |  | ESR1 | 0.67 |  |
| 9 |  | CDK6 | 0.701 |  |
| 10 |  | EZH2 | 0.779 |  |
| 11 |  | E2F3 | 0.807 |  |
| 12 | CCNE1 | PGR | 0.415 | 15 |
| 13 |  | CHAF1A | 0.489 |  |
| 14 |  | EZH2 | 0.52 |  |
| 15 |  | ESR1 | 0.521 |  |
| 16 |  | KPNA2 | 0.528 |  |
| 17 |  | PARP1 | 0.531 |  |
| 18 |  | PLK1 | 0.538 |  |
| 19 |  | SMARCA4 | 0.695 |  |
| 20 |  | FOXM1 | 0.776 |  |
| 21 |  | CDKN2A | 0.814 |  |
| 22 |  | E2F3 | 0.831 |  |
| 23 |  | PCNA | 0.896 |  |
| 24 |  | E2F2 | 0.908 |  |
| 25 |  | SKP2 | 0.99 |  |
| 26 |  | CDK6 | 0.999 |  |
| 27 | CDK6 | E2F3 | 0.832 | 12 |
| 28 |  | SMARCA4 | 0.424 |  |
| 29 |  | H2AX | 0.426 |  |
| 30 |  | PLK1 | 0.525 |  |
| 31 |  | ESR1 | 0.585 |  |
| 32 |  | PARP1 | 0.625 |  |
| 33 |  | FOXM1 | 0.667 |  |
| 34 |  | EZH2 | 0.686 |  |
| 35 |  | E2F2 | 0.776 |  |
| 36 |  | SKP2 | 0.806 |  |
| 37 |  | PCNA | 0.981 |  |
| 38 |  | CDKN2A | 0.999 |  |
| 39 | CDKN2A | SFRP1 | 0.53 | 18 |
| 40 |  | FOXA1 | 0.453 |  |
| 41 |  | E2F3 | 0.666 |  |
| 42 |  | SKP2 | 0.8 |  |
| 43 |  | H2AZ1 | 0.627 |  |
| 44 |  | PLK1 | 0.48 |  |
| 45 |  | H2AZ2 | 0.526 |  |
| 46 |  | EZH2 | 0.813 |  |
| 47 |  | PGR | 0.572 |  |
| 48 |  | ERBB4 | 0.56 |  |
| 49 |  | FOXM1 | 0.526 |  |
| 50 |  | SMARCA4 | 0.811 |  |
| 51 |  | E2F2 | 0.648 |  |
| 52 |  | PARP1 | 0.597 |  |
| 53 |  | PCNA | 0.448 |  |
| 54 |  | GATA3 | 0.646 |  |
| 55 |  | ESR1 | 0.667 |  |
| 56 |  | H2AX | 0.768 |  |
| 57 | CHAF1A | SKP2 | 0.544 | 4 |
| 58 |  | EZH2 | 0.407 |  |
| 59 |  | H2AX | 0.509 |  |
| 60 |  | PCNA | 0.898 |  |
| 61 | CXXC5 | ESR1 | 0.786 | 1 |
| 62 | E2F3 | E2F3 | 0.962 | 6 |
| 63 |  | SKP2 | 0.653 |  |
| 64 |  | PLK1 | 0.474 |  |
| 65 |  | EZH2 | 0.718 |  |
| 66 |  | FOXM1 | 0.54 |  |
| 67 |  | KPNA2 | 0.455 |  |
| 68 |  | H2AX | 0.405 |  |
| 69 |  | SKP2 | 0.427 | 6 |
| 70 |  | FOXM1 | 0.435 |  |
| 71 |  | PCNA | 0.455 |  |
| 72 |  | PLK1 | 0.456 |  |
| 73 |  | EZH2 | 0.732 |  |
| 74 | ERBB4 | SHC2 | 0.958 | 4 |
| 75 |  | EZH2 | 0.412 |  |
| 76 |  | PGR | 0.881 |  |
| 77 |  | ESR1 | 0.961 |  |
| 78 | ESR1 | SFRP1 | 0.433 | 16 |
| 79 |  | FOXA1 | 0.998 |  |
| 80 |  | RARA | 0.699 |  |
| 81 |  | SKP2 | 0.554 |  |
| 82 |  | H2AZ1 | 0.645 |  |
| 83 |  | PLK1 | 0.42 |  |
| 84 |  | H2AZ2 | 0.535 |  |
| 85 |  | EZH2 | 0.915 |  |
| 86 |  | PGR | 0.974 |  |
| 87 |  | FOXM1 | 0.79 |  |
| 88 |  | SMARCA4 | 0.859 |  |
| 89 |  | PARP1 | 0.746 |  |
| 90 |  | GATA3 | 0.982 |  |
| 91 |  | PPARA | 0.618 |  |
| 92 |  | KPNA2 | 0.596 |  |
| 93 |  | H2AX | 0.681 |  |
| 94 | EZH2 | SFRP1 | 0.4 | 15 |
| 95 |  | FOXA1 | 0.492 |  |
| 96 |  | RARA | 0.866 |  |
| 97 |  | SKP2 | 0.415 |  |
| 98 |  | H2AZ1 | 0.784 |  |
| 99 |  | PLK1 | 0.508 |  |
| 100 |  | H2AZ2 | 0.687 |  |
| 101 |  | KPNA2 | 0.411 |  |
| 102 |  | PCNA | 0.419 |  |
| 103 |  | PGR | 0.432 |  |
| 104 |  | PARP1 | 0.592 |  |
| 105 |  | GATA3 | 0.672 |  |
| 106 |  | FOXM1 | 0.778 |  |
| 107 |  | H2AX | 0.828 |  |
| 108 |  | SMARCA4 | 0.909 |  |
| 109 | FOXM1 | SFRP1 | 0.524 | 8 |
| 110 |  | FOXM1 | 0.423 |  |
| 111 |  | H2AZ2 | 0.561 |  |
| 112 |  | H2AX | 0.668 |  |
| 113 |  | H2AZ1 | 0.69 |  |
| 114 |  | RARA | 0.784 |  |
| 115 |  | PGR | 0.887 |  |
| 116 |  | GATA3 | 0.997 |  |
| 117 |  | SKP2 | 0.592 | 6 |
| 118 |  | PLK1 | 0.995 |  |
| 119 |  | GATA3 | 0.45 |  |
| 120 |  | PARP1 | 0.454 |  |
| 121 |  | KPNA2 | 0.632 |  |
| 122 |  | SMARCA4 | 0.75 |  |
| 123 | GATA3 | RARA | 0.786 | 6 |
| 124 |  | H2AZ1 | 0.612 |  |
| 125 |  | H2AZ2 | 0.557 |  |
| 126 |  | PGR | 0.81 |  |
| 127 |  | SMARCA4 | 0.617 |  |
| 128 |  | H2AX | 0.539 |  |
| 129 | H2AX | RARA | 0.556 | 9 |
| 130 |  | H2AZ1 | 0.721 |  |
| 131 |  | PLK1 | 0.745 |  |
| 132 |  | PYGO1 | 0.544 |  |
| 133 |  | H2AZ2 | 0.636 |  |
| 134 |  | PGR | 0.444 |  |
| 135 |  | SMARCA4 | 0.876 |  |
| 136 |  | PARP1 | 0.964 |  |
| 137 |  | KPNA2 | 0.597 |  |
| 138 | H2AZ1 | RARA | 0.587 | 8 |
| 139 |  | H2AZ2 | 0.478 |  |
| 140 |  | PYGO1 | 0.546 |  |
| 141 |  | PARP1 | 0.617 |  |
| 142 |  | PLK1 | 0.674 |  |
| 143 |  | KPNA2 | 0.78 |  |
| 144 |  | PCNA | 0.812 |  |
| 145 |  | SMARCA4 | 0.831 |  |
| 146 |  | RARA | 0.656 | 6 |
| 147 |  | PLK1 | 0.621 |  |
| 148 |  | PYGO1 |  |  |
| 149 |  | SMARCA4 | 0.422 |  |
| 150 |  | KPNA2 | 0.664 |  |
| 151 |  | PCNA | 0.799 |  |
| 152 | KPNA2 | SKP2 | 0.489 | 5 |
| 153 |  | PLK1 | 0.508 |  |
| 154 |  | SMARCA4 | 0.689 |  |
| 155 |  | PARP1 | 0.52 |  |
| 156 |  | PCNA | 0.527 |  |
| 157 | PARP1 | RARA | 0.521 | 6 |
| 158 |  | SKP2 | 0.457 |  |
| 159 |  | PLK1 | 0.561 |  |
| 160 |  | PGR | 0.711 |  |
| 161 |  | SMARCA4 | 0.996 |  |
| 162 |  | PCNA | 0.872 |  |
| 163 | PCNA | PLK1 | 0.431 | 1 |
| 164 | PGR | PPARA | 0.461 | 2 |
| 165 |  | SMARCA4 | 0.586 |  |
| 166 | PLK1 | SKP2 | 0.628 | 1 |

**Supplementary material 1 References**

3. Miranda F, Prazeres H, Mendes F, Martins D, Schmitt F. Resistance to endocrine therapy in HR + and/or HER2 + breast cancer: the most promising predictive biomarkers. Mol Biol Rep. (2022), 49:717–33. doi: 10.1007/s11033-021-06863-3

11. Sanders DA, Ross-Innes CS, Beraldi D, Carroll JS, Balasubramanian S. Genome-wide mapping of FOXM1 binding reveals co-binding with estrogen receptor alpha in breast cancer cells. Genome Biol. (2013) 24;14(1):R6. doi: 10.1186/gb-2013-14-1-r6

31. Zhou W, Srinivasan S, Nawaz Z, Slingerland JM. ERa, SKP2 and E2F-1 form a feed forward loop driving late ERa targets and G1 cell cycle progression. Oncogene. (2014) 33:2341–53. doi: 10.1038/onc.2013.19732

32. Wierer M, Verde G, Pisano P, Molina H, Font-Mateu J, DiCroce L, et al. PLK1 signaling in breast cancer cells cooperates with estrogen receptor-dependent gene transcription. Cell Rep. (2013) 3:2021–32. doi: 10.1016/j.celrep.2013.05.024

34. Wu Y, Zhang Z, Cenciarini ME, Proietti CJ, Amasino M, Hong T, et al. Tamoxifen resistance in breast cancer is regulated by the EZH2–ERa–GREB1 transcriptional axis. Cancer Res. (2018) 78:671–84. doi: 10.1158/0008-5472.CAN-17-1327

37. Millour J, De Olano N, Horimoto Y, Monteiro LJ, Langer JK, Aligue R, et al. ATM and p53 regulate FOXM1 expression via E2F in breast cancer epirubicin treatment and resistance. Mol Cancer Ther. (2011) 10:1046–58. doi: 10.1158/1535-7163.MCT-11-0024

50. Wei L, Bai Y, Na L, Sun Y, Zhao C, Wang W. E2F3 induces DNA damage repair, stem-like properties and therapy resistance in breast cancer. Biochim Biophys Acta Mol Basis Dis. (2023) 1869(8):166816. doi: 10.1016/j.bbadis.2023.166816

51. Zhao W, Rose SF, Blake R, Godicelj A, Cullen AE, Stenning J, et al. ZMIZ1 enhances ERa-dependent expression of E2F2 in breast cancer. J Mol Endocrinol. (2024) 73(1):e230133. doi: 10.1530/JME-23-013360

60. Takeiwa T, Ikeda K, Suzuki T, Sato W, Iino K, Mitobe Y, et al. PSPC1 is a potential prognostic marker for hormone-dependent breast cancer patients and modulates RNA processing of ESR1 and SCFD2. Sci Rep. (2022) 12:9495. doi: 10.1038/s41598-022-13601-7

61. Xia X, Liao Y, Guo Z, Li Y, Jiang L, Zhang F, et al. Targeting proteasome-associated deubiquitinases as a novel strategy for the treatment of estrogen receptor-positive breast cancer. Oncogenesis. (2018) 7:75. doi: 10.1038/s41389-018-0086-y

65. Clemenceau A, Lacouture A, Bherer J, Ouellette G, Michaud A, Audet-Walsh É, et al. Role of secreted frizzled-related protein 1 in early breast carcinogenesis and breast cancer aggressiveness. Cancers (Basel). (2023) 15:2251. doi: 10.3390/cancers15082251

66. Diep CH, Ahrendt H, Lange CA. Progesterone induces progesterone receptor gene (PGR) expression via rapid activation of protein kinase pathways required for cooperative estrogen receptor alpha (ER) and progesterone receptor (PR) genomic action at ER/PR target genes. Steroids. (2016) 114:48–58. doi: 10.1016/j.steroids.2016.09.004

67. Fu X, Pereira R, Liu CC, De Angelis C, Shea MJ, Nanda S, et al. High FOXA1 levels induce ER transcriptional reprogramming, a pro-metastatic secretome, and metastasis in endocrine-resistant breast cancer. Cell Rep. (2023) 42:112821. doi: 10.1016/j.celrep.2023.11282174

74. Takaku M, Grimm SA, De Kumar B, Bennett BD, Wade PA. Cancer-specific mutation of GATA3 disrupts the transcriptional regulatory network governed by estrogen receptor alpha, FOXA1 and GATA3. Nucleic Acids Res. (2020) 48:4756–68. doi: 10.1093/nar/gkaa179

75. Lin M, Wang YN, Ye Y, Xiong Z, Guo F, Chen H. DNAJC12 as a mediator between ESR1 and ERBB4 in breast carcinoma cells. Front Oncol. (2021) 11. doi: 10.3389/fonc.2021.582277

76. Makhlouf S, Almalki N, Sheha A, Atallah NM, Ibrahim A, Toss M, et al. Outcome prediction of oestrogen receptor-positive breast cancer based on a panel of oestrogen receptor-regulated genes. Histopathology. (2025) 87:842–55. doi: 10.1111/his.15557

77. Plaza-Menacho I, Morandi A, Robertson D, Pancholi S, Drury S, Dowsett M, et al. Targeting the receptor tyrosine kinase RET sensitizes breast cancer cells to tamoxifen treatment and reveals a role for RET in endocrine resistance. Oncogene. (2010) 29:4648–57. doi: 10.1038/onc.2010.209

78. Laganière J, Deblois G, Giguère V. Functional genomics identifies a mechanism for estrogen activation of the retinoic acid receptor 1 gene in breast cancer cells. Mol Endocrinol. (2005) 19:1584–92. doi: 10.1210/me.2005-0040

79. Faddy HM, Robinson JA, Lee WJ, Holman NA, Monteith GR, Roberts-Thomson SJ. Peroxisome proliferator-activated receptor a expression is regulated by estrogen receptor a and modulates the response of MCF-7 cells to sodium butyrate. Int J Biochem Cell Biol. (2006) 38:255–66. doi: 10.1016/j.biocel.2005.09.00280

80. Yaşar P, Ayaz G, Muyan M. Estradiol-estrogen receptor a mediates the expression of the CXXC5 gene through the estrogen response element-dependent signaling pathway. Sci Rep. (2016) 6:37808. doi: 10.1038/srep37808

81. Audero E, Cascone I, Maniero F, Napione L, Arese M, Lanfrancone L, et al. Adaptor ShcA protein binds tyrosine kinase Tie2 receptor and regulates migration and sprouting but not survival of endothelial cells. J Biol Chem. (2004) 279:13224–33. doi: 10.1074/jbc.M307456200

82. Frietze S, Lupien M, Silver PA, Brown M. CARM1 regulates estrogen-stimulated breast cancer growth through up-regulation of E2F1. Cancer Res. (2008) 68:301–6. doi: 10.1158/0008-5472.CAN-07-1983

83. Yang C, Li Z, Bhatt T, Dickler M, Giri D, Scaltriti M, et al. Acquired CDK6 amplification promotes breast cancer resistance to CDK4/6 inhibitors and loss of ER signaling and dependence. Oncogene. (2017) 36:2255–64. doi: 10.1038/onc.2016.379

84. Samman A, Hanbazazh M, Samargandy S, Al-Maghrabi J. Expression of p16 protein in breast cancer. Polish J Pathol. (2024) 75:19–24. doi: 10.5114/pjp.2024.136028

85. Bhatt S, Xiao Z, Meng Z, Katzenellenbogen BS. Phosphorylation by p38 mitogenactivated protein kinase promotes estrogen receptor a turnover and functional activity via the SCF Skp2 proteasomal complex. Mol Cell Biol. (2012) 32:1928–43. doi: 10.1128/mcb.06561-1186

86. Yang TL, Tsai CH, Su YW, Chang YC, Lee F, Huang TY, et al. Combining KPNA2 and FOXM1 expression as prognostic markers and therapeutic targets in hormone receptor-positive, HER2-negative breast cancer. Cancers (Basel). (2025) 17:671. doi: 10.3390/cancers17040671

87. Baron S, Escande A, Albérola G, Bystricky K, Balaguer P, Richard-Foy H. Estrogen receptor a and the activating protein-1 complex cooperate during insulin-like growth factor-I-induced transcriptional activation of the pS2/TFF1 gene. J Biol Chem. (2007) 282:11732–41. doi: 10.1074/jbc.M610079200

88. Sun X, Ma Q, Cheng Y, Huang H, Qin J, Zhang M, et al. Overexpression of CHAF1A is associated with poor prognosis, tumor immunosuppressive microenvironment and treatment resistance. Front Genet. (2023) 14. doi: 10.3389/fgene.2023.1108004

89. Srivastava N, Gochhait S, Gupta P, Bamezai RNK. Copy number alterations of the H2AFX gene in sporadic breast cancer patients. Cancer Genet Cytogenet. (2008) 180:121–8. doi: 10.1016/j.cancergencyto.2007.09.024

90. Gévry N, Hardy S, Jacques PÉ, Laflamme L, Svotelis A, Robert F, et al. Histone H2A.Z is essential for estrogen receptor signaling. Genes Dev. (2009) 23:1522–33. doi: 10.1101/gad.1787109

91. Loh YN, Hedditch EL, Baker LA, Jary E, Ward RL, Ford CE. The Wnt signalling pathway is upregulated in an in vitro model of acquired tamoxifen resistant breast cancer. BMC Cancer. (2013) 13:174. doi: 10.1186/1471-2407-13-174

92. Schultz-Norton JR, Gabisi VA, Ziegler YS, McLeod IX, Yates JR, Nardulli AM. Interaction of estrogen receptor a with proliferating cell nuclear antigen. Nucleic Acids Res. (2007) 35:5028–38. doi: 10.1093/nar/gkm533

93. Zhang Y, Wester L, He J, Geiger T, Moerkens M, Siddappa R, et al. IGF1R signaling drives antiestrogen resistance through PAK2/PIX activation in luminal breast cancer. Oncogene. (2018) 37:1869–84. doi: 10.1038/s41388-017-0027-9

94. Fizal NS, Hasnizan NYU, Chong CF, Chuan SK, Ahmed AF, Mokhtar AMA. Rho/PAK signaling: a key driver of anthracycline and taxane resistance in breast cancer. Med Oncol. (2026) 43:183. doi: 10.1007/S12032-026-03294-7

95. Li X, Wen W, Liu K, Zhu F, Malakhova M, Peng C, et al. Phosphorylation of caspase-7 by p21-activated protein kinase (PAK) 2 inhibits chemotherapeutic drug induced apoptosis of breast cancer cell lines. J Biol Chem. (2011) 286:22291–9. doi: 10.1074/jbc.M111.236596

96. Gadad SS, Camacho CV, Malladi V, Hutti CR, Nagari A, Lee Kraus W. Poly(ADPribose) polymerase-1 regulates estrogen-dependent gene expression in estrogen receptor a-positive breast cancer cells. Mol Cancer Res. (2021) 19:1688–98. doi: 10.1158/1541-7786.MCR-21-0103

97. Conger AK, Martin EC, Yan TJ, Rhodes LV, Hoang VT, La J, et al. Argonaute 2 expression correlates with a Luminal B breast cancer subtype and induces estrogenreceptor alpha isoform variation. Noncoding RNA. (2016) 2:8. doi: 10.3390/ncrna2030008

98. Elazezy M, Schwentesius S, Stegat L, Wikman H, Werner S, Mansour WY, et al. Emerging insights into keratin 16 expression during metastatic progression of breast cancer. Cancers (Basel). (2021) 13:3869. doi: 10.3390/cancers13153869
